# The *Drosophila* escape motor circuit shows differential vulnerability to aging linked to functional decay

**DOI:** 10.1371/journal.pbio.3003553

**Published:** 2025-12-16

**Authors:** Alexandros Gaitanidis, Veronica Pampanin, Jessica Thiem, Georgios Kalaras, Lion Huthmacher, Silvan Hürkey, Dimitrios Kadas, Agapi Dimitriadou, Andriana Ntogka, Subhabrata Sanyal, Christos Consoulas, Carsten Duch

**Affiliations:** 1 Laboratory of Experimental Physiology, Medical School, National and Kapodistrian University of Athens (NKUA), Athens, Greece; 2 Institute of Developmental Biologie and Neurobiology, Johannes Gutenberg University Mainz, Mainz, Germany; 3 Department of Translational Neuroscience, Calico Life Sciences LLC, San Francisco, California, United States of America; University of Michigan, UNITED STATES OF AMERICA

## Abstract

Brain aging can cause cognitive and motor disabilities which often correlate with changes in dendritic branch, axon collateral, and synapse numbers. However, from invertebrates to mammals, age-related decline is typically restricted to specific neuron types or brain parts, indicating differential vulnerability. The rules to pinpoint the susceptibility of distinct brain elements to aging remain largely unknown. Here, we combine longitudinal studies with neuroanatomical, electrophysiological, and optophysiological analyses in the *Drosophila* genetic model to identify aging-susceptible and aging-resilient elements in a sensorimotor circuit that underlies escape. Young and mid-aged flies escape predator-like visual stimuli with a jump followed by flight, but behavioral performance declines with age. Mapping the underlying functional decline into the brain shows that most circuit components are robust against aging and remain functional even in old flies that have lost the behavior. By contrast, behavioral decline is caused by the selective decay of synaptic transmission between one specific visual projection neuron type (LC4) and the dendrite of one identified descending neuron (GF). Structurally, presynaptic active zone marker density is reduced whereas postsynaptic marker density remains normal. Other central synapses in this circuit as well as neuromuscular synapses are robust to aging. The synaptic connection susceptible to aging is also the circuit element most vulnerable to starvation or oxidative stress. Moreover, the vulnerable circuit element is also required for habituation, and thus, underlying circuit plasticity. In conjunction with data from mammalian brains our data suggest that a trade-off for functional neural circuit plasticity might be vulnerability to aging.

## Abstract 2

Age-related decline is restricted to specific neuron types or brain regions, indicating they have different vulnerability; however how different brain components age remains unknown. This study shows that neural components of the Drosophila escape motor circuit show different susceptibilities to aging and acute stressors, with plastic components being vulnerable and hard-wired components robust.

## Introduction

Brain aging is accompanied by functional decline. Although it remains unclear whether aging mechanisms exist that are common to all brain cells, it has become clear that distinct brain compartments and/or specific neuron types are particularly vulnerable, both in the aging brains of vertebrates and invertebrates as well as in neurodegenerative diseases [1]. The core symptoms of Parkinsons disease, for example, are caused by the selective degeneration of dopaminergic neurons in the substantia nigra [2], whereas other neurodegenerative diseases, such as ALS, are characterized by motoneuron loss [3]. By contrast, in aging brains, rather than neuron loss, anatomical correlates of functional decline include alterations in the number of synaptic connections [4,5], dendritic tree length [6,7], axon collateral number, and myelination [8,9]. These changes are typically restricted to specific brain parts and neuron types, both in mammals [10–12] and in invertebrates [5,13–16]. However, it remains largely unknown how to predict which elements of neural circuitry are robust and which are vulnerable to aging.

Animal models, including invertebrates such as the nematode worm, *Caenorhabditis eleg ans*, and the fruit fly, *Drosophila melanogaster*, have helped identifying conserved molecular mechanisms that contribute to aging [17,18]. For example, combining motor performance assays with genetics in *C.elegans* has helped linking molecular pathways, such as insulin/insulin-like signaling (IIS), to age-related transitions between health-span, mid-life disability, and late-life frailty [19]. Insulin signaling is also a major factor of aging in mammals [20,21] and in fruit flies [22], thus indicating that some mechanisms of aging are highly conserved. In *Drosophila* genetic, pharmacological, and dietary manipulations have been reported to prolong life span [22] and/or to postpone age-related motor [23–25] and cognitive decline [13,26]. In some cases, this has allowed to identify age-related molecular and structural changes of central nervous synapses that underlie behavioral decline [27,28] and seem to be conserved from flies to mammals [29,30]. However, precise mapping of age-related performance defects onto brain circuitry remains a challenge, and it is largely unclear whether and why different circuit components age differently.

Here, we utilize experimental advantages of *Drosophila* to identify the neural substrate causing the late-life loss of an innate behavior, the escape motor response. Escape is evolutionarily relevant, can be readily assayed, and the underlying neural circuit is well described, down to all component neurons and synaptic connections [31–33]. When confronted with a potentially life-threatening visual stimulus, flies escape by initiating a jump followed by flight. We find that escape responsiveness declines in old flies and map the underlying neural cause. Surprisingly, most circuit components are extremely robust against aging. By contrast, age-related behavioral decline can be traced down to one specific plastic synapse in the circuit. This supports the view that the price for functional plasticity is an increased vulnerability to aging.

## Results

### Flies fail to engage into visually induced escape at very old ages

**Fig 1:**
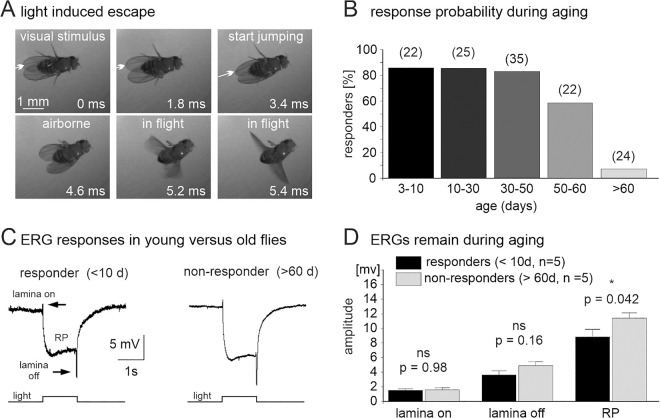
Age-related decline of *Drosophila* innate escape behavior. **(A)** Snapshots from a high-speed video (5,000 fps taken under UV illumination) of a fly during a typical visually induced escape motor response (jumping followed by flying). Escape can be induced by visual stimuli, such as a 20 ms light-off stimuli (light induced escape response, LIER, see Methods). Selected snapshots show the fly during the white light-off stimulus (top left), just after the light stimulus (top middle), during the start of the jump (top right) as indicated by the first forward movement of the body (white arrows in top row), just airborne (bottom left), airborne with the wings fully extended and beating (bottom middle and bottom right). **(B)** The percentage of animals that reliably respond with escape to a light-off stimulus is above 80% in young (3–10 days) and mid-aged animals (10–30 and 30–50 days), but decreases markedly in old (50–60 days) and very old animals (older than 60 days). Number of animals tested per age group in brackets. **(C)** Representative electroretinogram (ERG) recordings reveal no obvious differences between young animals that exhibit light-induced escape (responders younger than 10 days) and old animals (>60 days) that do not exhibit light-induced escape (NRs). **(D)** Quantification reveals a significant difference of the amplitudes of the photoreceptor potential (RP, *p = 0.042) but not the lamina on (*p* = 0.98) and lamina off transients (*p* = 0.16) between young responders (black bars) and old NRs (gray bars). Bars are means and error bars standard errors of the mean (*p* values from two-sided unpaired Student’s *t* test. See S1 Data for individual data points.

Escape responses can robustly be induced by visual stimulation, either by looming stimuli [33], or by dark flashes of 20 ms duration under bright light ambient illumination (for details see Methods). Fig 1A shows representative images of a high-speed video taken from a typical dark flash induced escape response. No movement is observed during roughly the first 2 ms after the visual stimulus. The first forward movement is observed after 3.4 ms (Fig 1A, see white arrow), and after roughly 4.5 ms the animal becomes airborne, though the wings are not yet fully extended. Full wing extension and beating of the wings starts within another milliseconds after take-off. Extensive description and quantification of this escape behavior in response to light-off [34,35] as well as in response to looming stimuli [36] has previously been conducted and is not repeated in this study, which focusses on escape behavior and circuit aging. Behavioral testing of adult flies reveals that escape is robustly induced in >80% of the animals tested at young (3–10 days), mid (10–30 days), and mid-to-old ages (30–50 days). There are no significant differences in response probability up to the 50th day of life (Fig 1B). By contrast, in aged flies (50–60 days), response probability drops to below 60%, and to below 10% in flies older than 60 days (Fig 1B).

### Neither phototransduction nor synaptic transmission to first order visual interneurons cause the late-life loss of escape

One possible cause for the observed >8-fold decline in responsiveness in old flies could be an inability to detect the visual stimulus. We employ electroretinograms (ERGs) to probe for visual responsiveness in the different age groups. ERGs show a compound field potential that results from light induced photoreceptor excitation and synaptic transmission between photoreceptors and lamina first order visual interneurons [37,38]. *Drosophila* ERGs show 3 distinct components (Fig 1C): The receptor potential (RP) corresponds inversely to the sum of the light induced depolarization of all photoreceptors. The lamina on transient reflects chloride influx induced hyperpolarization in lamina interneurons as induced by histaminergic synaptic transmission from photoreceptors to lamina 1 and 2 interneurons [39]. The lamina off response reflects lamina interneuron depolarization as resulting from the termination of histamine release from photoreceptors [40]. Although ERGs will not reveal subtle alterations in phototransduction or visual processing, they are a reliable measure of whether phototransduction functions in R1–R6 and whether photoreceptor excitation is synaptically transmitted to first order visual interneurons. Given that we induce escape with light on-off stimuli, intricate aspects of visual processing, such as shape, edge, and movement detection are unlikely required, making the ERG a useful measure for basic visual function during circuit aging. Importantly, young animals (<10 days) that display escape responses in behavioral tests (responders) and old animals (>60 days) that fail to escape upon visual stimulation display similar ERGs (Fig 1C and 1D). Only the amplitude of the sum photoreceptor potential (RP) displays a significant difference with amplitudes being significantly larger in old nonresponders (NRs) (Fig 1D). We judge this increase insignificant in this context as initial visual processing is obviously intact. The amplitudes of the lamina on and the lamina off responses show no differences between the groups (Fig 1C and 1D). In sum, reduced phototransduction or synaptic transmission to first order visual interneurons is unlikely the cause for the observed aging-related decline in escape motor behavioral responses. Therefore, if aging-related neural defects underlie behavioral decline in old animals, these occur downstream of retina and lamina in the escape circuit.

### The neural circuit underling visually induced escape

**Fig 2:**
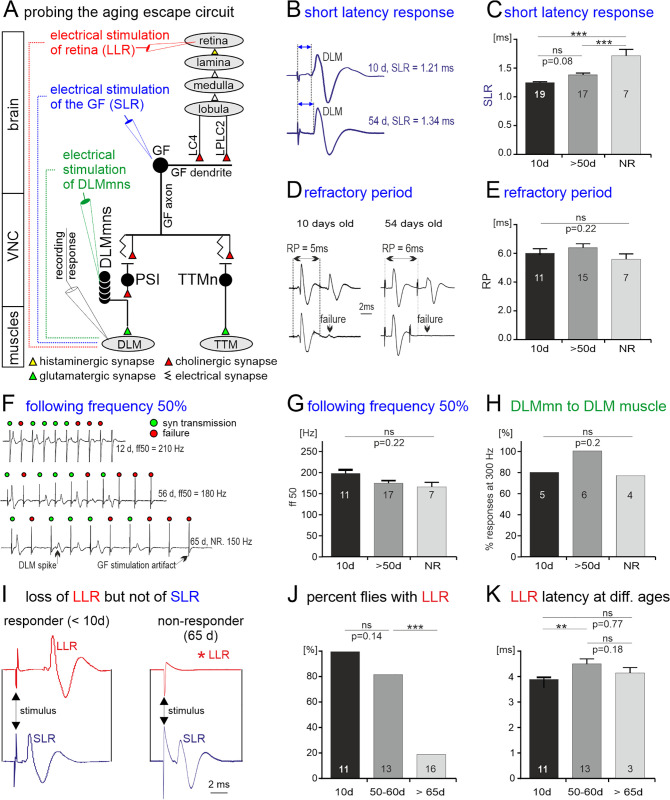
Probing the neuronal escape circuit for age-related functional decline. **(A)** Schematic circuit diagram. Visual input is computed in four visual neuropils (retina, lamina, medulla, and lobula) and relayed via the lobular columnar type 4 (LC4) and the lobula plate/lobula columnar, type 2 (LPLC2) interneurons onto the dendrites of the descending giant fiber neuron (GF). In the ventral nerve cord (VNC) the GF axon terminals connect with mixed electrical/chemical synapses to the jump motoneuron (TTM Mn) and to the PSI interneuron (PSI). The PSI in turn forms cholinergic synapses onto the motoneurons (DLM Mns) to the DLM flight muscle. Circuit output is assayed by recording firing output from fiber 6 of the dorsal longitudinal flight muscle (DLM, black electrode). Each DLM spike reflects one spike of the DLM Mn innervating this fiber. Circuit output can be evoked either by extracellular stimulation of retina (red electrode) which yields a long latency response (LLR, red) in the DLM, or by stimulation of the giant fiber (GF) descending neuron (blue electrode), thus bypassing all visual brain circuitry, which yields a short latency response (SLR blue). Neuromuscular transmission can also be assayed by direct stimulation of the DLM Mns (green electrode) with simultaneous DLM recording (black electrode). **(B)** Representative traces of SLR DLM responses to GF stimulation in a young (top) and an old animal (bottom). **(C)** SLR increases mildly during aging and significantly in nonresponders (NR), but SLR is reliably evoked at all ages, even in old NRs (ANOVA with Tukey’s multiple comparisons). **(D, E)** Refractory period of the SLR shows no changes with aging or in NRs (ANOVA). **(F, G)** The rate with which 50% of all GF spikes are reliably transmitted through the downstream circuit (following frequency 50%) does not decrease significantly with aging (ANOVA). **(H)** Synaptic transmission frequencies of 300 Hz at the synapse from flight motoneurons to the DLM are retained with aging and remain even in NRs (Fisher’s exact test). **(I)** Representative traces showing LLR (red) upon retina stimulation and SLR (blue) upon GF stimulation in a young (left traces) vs. an old nonresponding animal (right). **(J)** Quantification reveals that LLR is reliably evoked in young animals, but the percentage of animals that do not show LLR increases significantly in old flies (Fisher’s test). **(K)** For LLR the signal transmission speed through the circuit decreases mildly during mid-life, but is not significantly different between young and old animals (ANOVA with Tukey’s test). See S1 Data for individual data points.

The *Drosophila* optic lobe consists of four main parts [41]. Retinal photoreceptors 1–6 connect to first order interneurons in lamina. Lamina interneurons terminate in the medulla, together with photoreceptors 7 and 8 from retina. Downstream of medulla are lobula and lobula plate, where visual information relevant to escape is relayed to lobular columnar type 4 (LC4) and to lobular plate/lobular columnar type 2 (LPLC2) visual projection neurons (Fig 2A). Both LC4 and LPLC2 provide major synaptic input to the giant fiber descending neuron (GF) dendrite [33,42,43] and make up 98.5% of the GF’s direct input synapses from the optic lobe [44]. LC4 encodes information about velocity of the visual stimulus, and optogenetic activation of LC4 elicits fast escape as mediated by GF activation [32]. LPLC2 encodes information about the size component of the visual stimulus and is required for looming induced fast escape responses mediated by GF activation [33]. The giant fiber (GF) descending neuron thus receives visual information to its dendrites located in the brain and relays this information through a descending axon to two thoracic motor sub-circuits (Fig 2A): the GF-TTM (tergotrochanteral muscle) system, controlling the extension of mesothoracic legs during escape jumping, and the GF-DLM (dorsal longitudinal flight muscle) system, controlling wing downstroke during flight (Fig 2A). In the GF-TTM and GF-DLM pathways, the GF makes mixed electrical and chemical (cholinergic) synapses with the TTM jumping motoneuron (TTM Mn) and the peripherally synapsing interneuron (PSI) [45–49]. The PSI synapses directly onto the axons of the five motoneurons (DLM Mns) that innervate the DLM. The GF to DLM flight muscle pathway comprises one synapse (PSI-to-DLM Mns) more than the GF to TTM pathway (Fig 2A), thus adding the time for one chemical synaptic transmission onto the signal delay between GF and DLM muscle. Therefore, the typical GF mediated fast escape response is a jump followed by flight. Selected snapshots from a high-speed video (5,000 fps) of a representative visually induced escape response show a jump within 3–4 ms after the visual stimulus followed by wing extension and flapping with an additional 1–2 ms delay (Fig 1A).

Output from the escape circuit cannot only be observed behaviorally, but it can also be recorded extracellularly in intact but restrained animals from the DLM by insertion of sharpened tungsten wires (Fig 2A). Importantly, the circuit can be stimulated at different levels. First, low amplitude electrical stimulation with a tungsten electrode inserted into retina causes excitation of photoreceptors and potentially also first order visual interneurons in lamina. This causes a so-called long latency response (LLR, ~5 ms latency) in the DLM fibers (Fig 2A, red). Second, by increasing electrical stimulation amplitude, the large GF descending neuron can be directly stimulated, thus bypassing the optic lobe and synaptic input integration in the GF. Accordingly, this causes a so-called short latency response (SLR, ~1.4 ms latency) in the DLM muscle (Fig 2A, blue). Importantly, recording the LLR from the DLM is a well-established read-out for circuit activation at the level of the optic lobe, whereas recording the SLR from the DLM is a well-established read-out for circuit activation at the level of the GF [50,51]. Although the extracellular stimulation always bears the danger of stimulating alternative circuit routes (see Methods and below), the specific latencies that we observe for SLR and LLR match precisely those that have been assigned to either optic lobe (LLR) or GF stimulation (SLR), and additional analyses further support this (see below). And third, insertion of the stimulation electrode directly into the ventral nerve cord allows for direct stimulation of the DLM Mns, thus bypassing all central circuitry. Comparing the effects of selective stimulation of the escape circuit at different levels in young animals that respond to visual stimuli with those in aged nonresponding animals can be used to narrow down the locus of aging-related circuit defects.

### Localizing the neural correlates for aging-related loss of escape behavior

Direct electrical stimulation of the GF descending neuron bypasses the optic lobes as well as the LC4 and LPLC2 visual projection neurons and evokes a short latency response (SLR, Fig 2A–2C). Although the delay of information transfer between the GF descending neuron and the flight muscle fibers increases with age (Fig 2C), SLR is reliably evoked throughout adult life, independent of age and of whether escape behavior is still exhibited by the animals. All 19 animals tested at the age of 10 days, as well as all 26 animals tested at ages above 50 days show SLR, independent of whether an old animal responds in behavioral testing (responder, *n* = 17) or not (NR, *n* = 7). The increase in SLR latency is reportedly caused by an age-related decrease in gap junction density at the GF output synapses in the thorax [52], but this increase in response latency by ~0.2 ms cannot account for the observed loss of escape response in old animals (see Discussion). This indicates that the reliability of information transfer through escape circuit elements downstream of spike initiation in the GF descending neuron is unlikely affected by aging. At all ages, the minimal temporal delay with which two consecutive GF stimuli are transmitted through the circuit (refractory period) is statistically similar in young and old flies (Fig 2D and 2E) as well as in old flies that show no behavioral escape response to visual stimulation (Fig 2E). Similarly, the maximum frequency at which 50% of the GF spikes in a train are successfully transmitted to the DLM fiber (Fig 2F, following frequency 50%) is ~180 Hz and similar in young responders and old NRs (Fig 2G). Finally, direct electrical stimulation of the DLM Mns is highly reliable for stimulation frequencies of up to 300 Hz through adult life, even in NRs (Fig 2H). Together, these data indicate that the GFS circuit elements downstream of spike initiation in the GF may be resilient to aging (but see also Fig 4 below).

**Fig 3:**
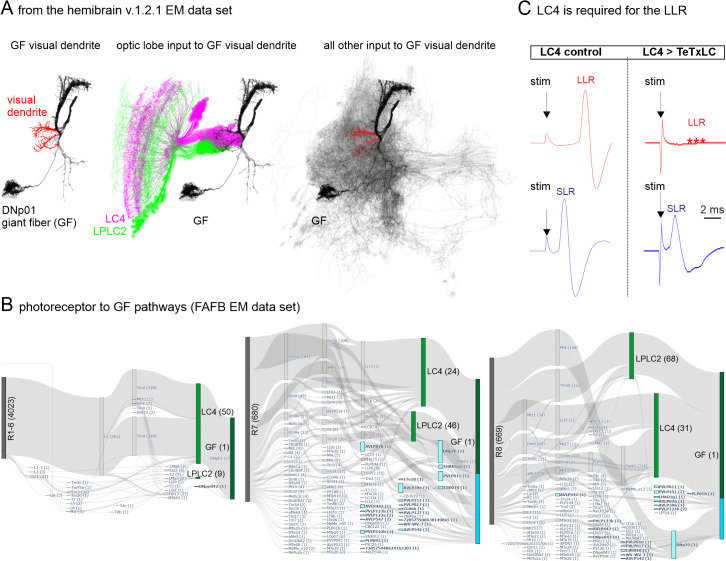
LC4 is required for the long latency response (LLR). **(A)** Reconstruction of the giant fiber descending neuron (GF or DNp01 v.1.2.1 hemibrain EM dataset) with its visual dendrite labeled red (left). Within the v.1.2.1 hemibrain connectome, 97.5% of the neurons that project to the GF (black) and receive input within lobula, lobula plate and (accessory) medulla are LC4 and LPLC2 neurons (LC4 (45.2%): magenta, LPLC2 (52.2%): green) (middle). Although LPLC2 and LC4 provide almost all input from the optic lobe to the GF, they make up only 62% of all inputs to the GF visual dendrites (red). The remaining 38% of inputs to the GF are provided by neurons that do not receive input within the optic lobe (gray, right). **(B)** Shortest path connectivity data of all photoreceptors to the GF within the right brain hemisphere derived from the FAFB connectome (R1–R6, left; R7, middle, R8 right). Each edge represents a connection of at least 5 synapses. Edge thickness represents total counts of individual photoreceptor paths traversing the respective edge. Node labels represent cell types with numbers of individual cells of each type in brackets. LC4 and LPLC2 are colored in green and the fraction of pathways in which LC4 and LPLC2 are the final node projecting to the GF is depicted in dark green. All other cell types that connect the photoreceptors indirectly to the GF are colored cyan and their sum fraction in blue. (**B**, left) 98.4% of R1-6 photoreceptors are connected to the GF via LC4 within 4 steps. (**B**, middle) 41.3% of R7 photoreceptors are connected to the GF via LC4, 16.2% via LPLC2 and 42.5% via other cell types. (**B**, right) 50.4% of R8 photoreceptors are connected to the GF via LC4, 25.1% via LPLC2 and 24.5% via other cell types. **(C)** Extracellular stimulation in the eyes as used to evoke SLR (blue) and LLR (red) throughout the study in a control (left) and upon silencing LC4 output by targeted expression of tetanus toxin light chain (TeTxLC). Expression of TeTxLC in LC4 eliminates LLR but does not affect SLR.

By contrast, in old animals that do not exhibit light induced escape behavior (NRs) electrical stimulation of retina evokes no long latency response (LLR) in the DLM fiber (red, Fig 2I), although SLR as induced by GF stimulation is normal (blue; Fig 2I). Quantification reveals that LLR is reliably evoked in all young animals (*n* = 11), only in about 75% of animals aged between 50 and 60 days (*n* = 13), and in less than 20% of the animals older than 65 days (*n* = 16; Fig 2J). In fact, the timing of age-related loss of LLR (Fig 2J) matches roughly that of age-related loss of escape behavior (Fig 1), indicating that the loss of LLR in the GF circuit is a good indicator for behavioral decline. In fact, old animals that do not show escape behavior anymore also do not show LLR in subsequent electrophysiological experiments. However, these animals still show SLR. By contrast, age-matched animals that still show the behavior exhibit both SLR and LLR in subsequent electrophysiological experiments. Interestingly, in those old animals that still show the behavior and exhibit LLR, latency is not different from that in young animals, indicating a sudden loss of function rather than a gradual worsening (Fig 2K). In sum, physiological analyses of the escape circuit at different levels reveal that age-related behavioral loss coincides with a loss of LLR.

**Fig 4:**
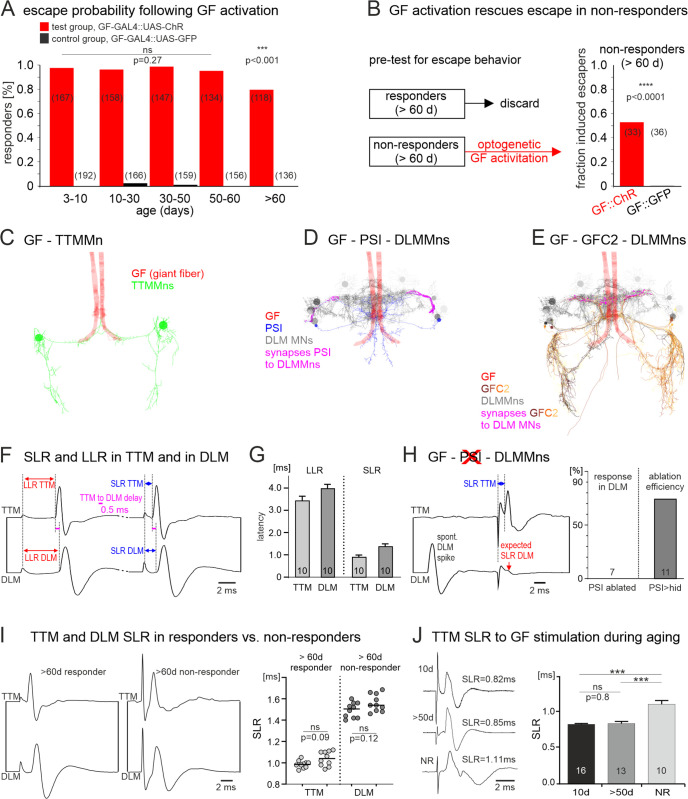
Optogenetic and electrical stimulation of the GF induces escape as well as SLR in the TTM and the DLM. **(A)** Optogenetic activation following targeted expression of UAS-CsChrimson only in the GF descending neuron reliably induces escape behavior until late life (red bars). Up to the age of 60 days no significant differences are observed in response probability (Fisher’s test). In animals older than 60 days, response percentage is significantly reduced (Fisher’s test) but still close to 80%. Escape is not evoked by identical red-light stimuli in control animals with targeted expression of UAS-GFP in the GF descending neuron. **(B)** A behavioral assay is used to select aged animals (>60 days) with UAS-CsChrimson expression in the GF descending neuron that fail to respond to visual stimulation with escape behavior (NRs). Optogenetic activation of the GF induced escape in nearly 50% of these pre-selected nonresponding animals (*n* = 33 animals) but in none of the 36 control animals with UAS-GFP instead of CsChrimson expression in the GF descending neuron. Fisher’s test reveals a highly significant difference between the test and the control group. **(C–E)** Neuron reconstructions of VNC members of the giant fiber escape circuit as extracted from the MANC connectome [53]. Bilateral GF projections (red) innervate the TTM Mns directly (**C**, green) and the DLM Mns (**D**, gray) via the PSIs (**D**, blue). All PSI output synapses to DLM Mns (n ~ 1,000) are localized along the axons of the DLM Mns (**D**, magenta). **(E)** Possible alternative pathway from the GF to the DLM Mns via the GF coupled neurons 2 (GFC2, yellow, see also [54]). GFC2 provide a total of ~ 600 input synapses to the DLM Mn axons (magenta). (F–I) Dual recordings from the TTM (top trace) and a DLM muscle fiber (bottom trace) under different conditions. (F, G) Control recording showing a ~ 0.5 ms delay between the TTM and the DLM read-outs of LLR (red) and SLR (blue). (**H**) The SLR remains normal in TTM but is absent in the DLM upon genetic ablation of the PSI in ~75% tested animals (right, % response in DLM). A spontaneous DLM spike confirms functionality. (**I**) In both, the TTM and the DLM, the SLR remains in old (>60 days) responding and age-matched nonresponding animals that have lost the behavior and LLR. The 0.5 ms delay in the SLR between TTM and DLM responses is faithfully observed (right). Latencies between responder and NR do not differ significantly (Mann–Whitney Test, TTM: *p* = 0.09, DLM: *p* = 0.12). **(J)** SLR remains reliable in TTM throughout aging, but its duration increases by ~0.2–0.3 ms during the aging process. See S1 Data for individual data points.

**Fig 5:**
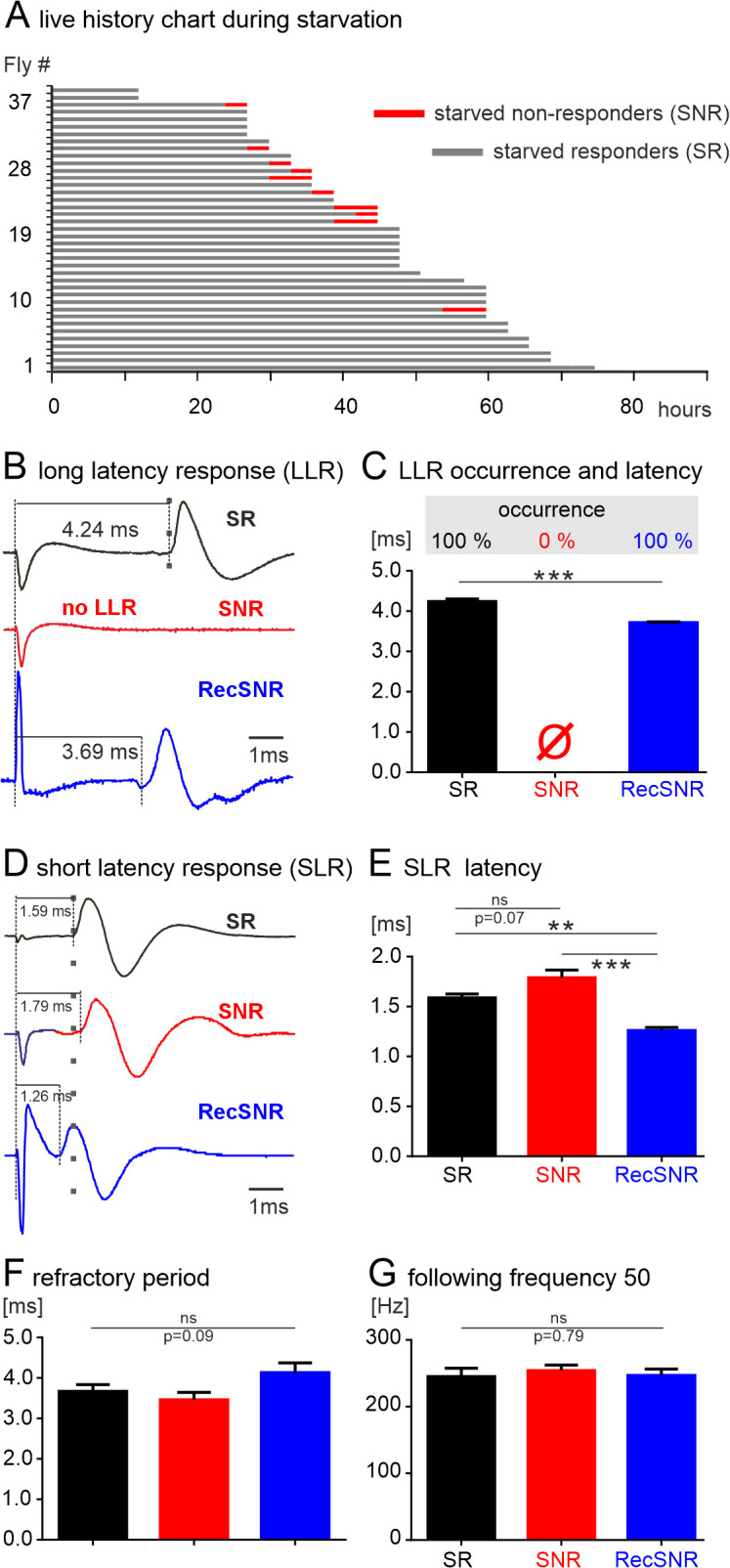
Probing the neuronal escape circuit for starvation induced functional decline. **(A)** Life history chart for 40 young (5 days old) flies that are continuously starved until death (or re-feeding, see below) and tested individually in the startle assay every 3 hours. Gray bars indicate the time span during starvation when the flies show climbing responses in the startle assay (starved responders; SR) and red bars indicate times when pre-death motor disabilities occur and starved flies fail to respond in the startle assay (starved NRs; SNR). **(B)** The lack of climbing responses in the startle assay goes along with the lack of the long latency response (LLR) in the giant fiber escape circuit. Representative DLM recordings in response to electrical stimulation of the compound eye in a starved responder (SR, black), a starved NR (SNR, red), and a starved NR that recovered from starvation induced motor disability through food supplementation (Rec SNR, blue). LLR is absent in starved NRs but regained after feeding. **(C)** Quantification of LLR measurements from animals of the three groups (SR black, SNR red, and Rec SNR blue, ***p < 0.0001, unpaired Student’s *t* test). **(D)** The short latency response (SLR) as evoked by electrical stimulation of the GF descending neuron is always present in all three test groups (SRs black, SNRs red, RecSNRs, blue). **(E)** SLR values are slightly but not significantly larger in starved NRs (red) than in control. Upon rescuing the starved NRs by feeding (RecSNR, blue), SLR duration is significantly reduced, even below the values of starved responders (SR, black). Between group comparisons by ANOVA with Tukey post hoc testing. **(F–G)** No significant differences (ANOVA) among the three groups were found with respect to the duration of the refractory period **(F)** or the stimulation frequency at which 50% of all GF descending neuron stimuli were successfully transmitted through the escape circuit to the DLM fibers **(G**, following frequency 50, FF50). See S1 Data for individual data points.

One concern with extracellular field potential stimulation is that it remains unclear how many and precisely which neurons are stimulated. The stimulation electrodes are inserted into the outer layer of the *Drosophila* optic lobe, the retina. Although we judge current spread to deeper optic lobe layers [41] or even to the central brain unlikely, we cannot exclude it. Bypassing LC4 and/or LPLC2 seems highly unlikely because these two types of visual projection neurons form 98.5% of all direct input synapses from the optic lobe to the GF visual dendrites as revealed by connectomics analyses of the hemibrain v1.2.1. EM dataset [44]. The GF with its visual dendrite and the respective projections from LC4 and LPLC2 are shown in Fig 3A. However, input synapses from optic lobe visual projection neurons makes only about two-thirds of all input to the GF visual dendrite, as it receives many additional inputs from other neurons (Fig 3A). Utilizing the FAFB EM data [55] to map the shortest pathways (4–5 synaptic hops) from each photoreceptor R1-6 to all GF dendrites reveals that 99.7% of pathways converge onto the GF through LC4 and LPLC2 (Fig 3B). Increasing pathway length to 6–7 synaptic hops still leaves 73.5% (for 6 hops) and 53.6% (for 7 hops) of pathways to terminate at the GF through LC4 and LPLCs. We estimate 7 hops to be the maximum path length in agreement with the ~3.4 ms LLR to the TTM if we assume a 0.5 ms latency of monosynaptic connections (PSI-to-DLM MN, Fig 4F). Although for R7 and R8 EM connectomics data also confirm LC4 and LPLC2 as the major optic lobe pathways to the GF, there are additional routes for information transfer to the GF at the level of shortest paths, for example, via the DNp70 descending neuron. However, this neuron is located outside the optic lobe in the central brain (Fig 3B).To experimentally exclude the possibility that extracellular stimulation in the eye bypasses the LC4 to GF synapse by either activating parallel pathways to the GF or by directly activating neurons even further downstream, we used the identical extracellular stimulation (0.1 ms duration and 8–10 V amplitude) as in all experiments (see Figs 2–5 and 8), but used *Drosophila* genetics to block synaptic transmission from LC4 to the GF dendrites by the selective expression of tetanus-toxins in LC4 neurons. If extracellular stimulation in the eye does not bypass LC4, this manipulation must take out the long latency response (LLR, that includes synaptic transmission delay from LC4 to the GF) but not the short latency response (direct stimulation of the GF). If the GF is not bypassed, the SLR should also retain the identical duration. This is precisely what we find (Fig 3C, *n* = 22 animals). By contrast, killing the GF by the targeted expression of UAS-hid (apoptosis signal, [56]) eliminates both LLR and SLR. Additional arguments against stimulating multiple parallel pathways are the highly reliable SLR and LLR values. For example, the LLR duration is at 3.92 ± 0.11 ms (mean and SD), and thus, shows a variation below 3% (variation coefficient = 0.028). The variance would be much higher if the extracellular stimulation would activate alternative neuronal routes that finally lead to the activation of the DLM. Together, these data validate previous [50,51,57,58] and our LLR and SLR read-outs of the responses to extracellular stimulation of the GFS either in the eye (LLR) or at the level of the GF (SLR).

**Fig 6:**
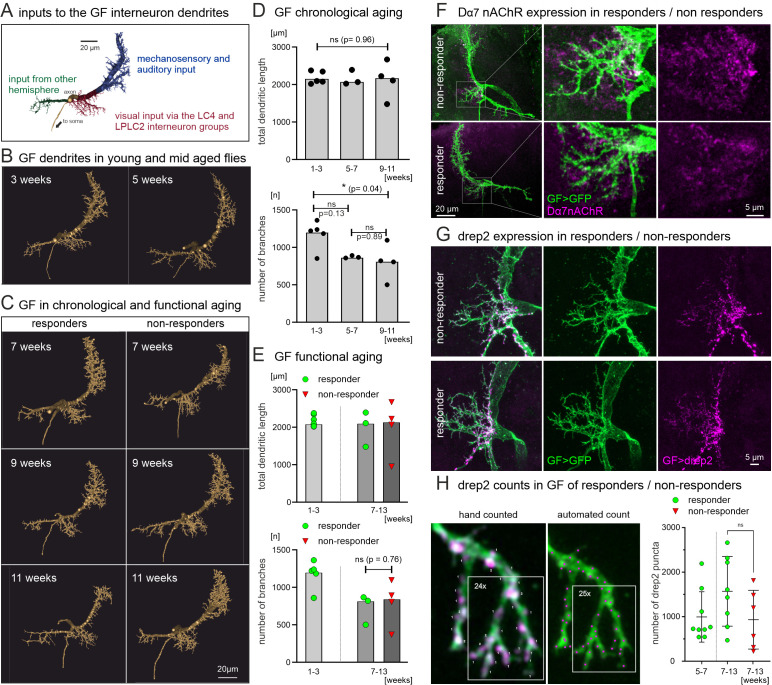
Chronological but not functional aging of GF descending neuron dendrite structure. **(A)** Representative 3-D reconstruction of GF dendritic structure color-coded for the different dendritic domains that receive input from different modalities. The visual input dendrite is coded in red. **(B)** Representative 3-D reconstruction of GF dendrites at the age of 3 (left) vs. 5 weeks (right). **(C)** Representative reconstruction of GF dendrites during chronological aging between weeks 7 and 11 from animals that show visually induced escape behavior (left column, responders) as compared to age-matched animas not showing visually induced escape behavior (right column, NRs). **(D)** Quantifying GF dendritic structure at different ages reveals no differences in total dendritic length (TDL, top diagram) between young (1–3 weeks), mid-aged (3–5 weeks), and old animals (9–11 weeks), but a significant decrease in the number of branches in old vs. young animals (bottom diagram). **(E)** By contrast, quantifying GF dendritic structure in old animals that respond with escape to visual stimulation (green circles) to animals that do not respond (red triangles) reveals no difference in total dendritic length (top diagram, ANOVA) or in the number of dendritic branches between old responders and old NRs (bottom diagram, unpaired Student’s *t* test). **(F)** Maximum intensity projection images of the GF descending neuron with UAS-GFP expression (green) and immunocytochemistry for Dα7nAChRs (magenta) in a representative 65 days old responder (bottom panels) vs. a representative 65 days old NR (top panels). Left panels show overview projection views of the CLSM image stacks and dotted white boxes demark the visual input area for which selective enlargements are shown in the middle panels (GF visual dendrites green, Dα7nAChRs magenta) and in the right panels (Dα7nAChRs only magenta). **(G)** Representative maximum intensity projection images of the GF descending neuron visual dendrite with UAS-GFP expression (green) and UAS-Drep2 expression (magenta) in 65 days old responder (bottom panels) vs. a 65 days old NR (top panels). Left panels show overlays, middle panels the GFP expression in the GF, and right panels the Drep2 expression in the GF. **(H)** Comparison of hand counted and automatically counted (see Methods) Drep2 puncta in a selective magnification of a part of the visual dendrite (left) shows comparable results (24 hand-counted vs. 25 automatically counted puncta). Quantification from mid-aged (5–7 weeks, green circles on the left) as well as old (7–13 weeks) responding (green circles in the center) and age-matched nonresponding animals (red triangles on the right) shows no significant difference. See S1 Data for individual data points.

**Fig 7:**
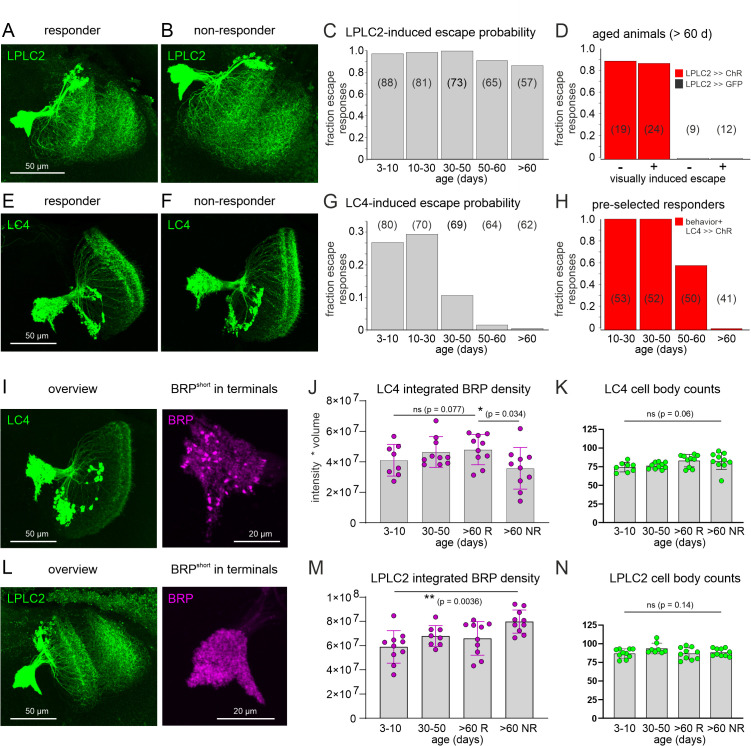
LC4 interneuron aging underlies escape circuit aging-related defects. **(A** and **B)** Maximum intensity projection views of representative CLSM image stacks of the LPLC2 interneuron in a >60 days old animal that responded to visual stimuli with escape behavior (responder, A) vs. in a >60 days old animal that did not respond to visual stimuli with escape behavior (NR, B) do not reveal massive reductions in LPLC2 arbor density or neuropil coverage. **(C)** In animals with selective expression of UAS-CsChrimson in LPLC2, optogenetic stimulation reliably induces escape responses through all age groups between 3 and 10 days and older than 60 days (>60 d) with no significant differences in the percentages between age groups (Fisher’s exact test, *p* = 0.051). **(D)** In animals >60 d, optogenetic activation of LPLC2 reliably induces escape behavior (response probability ~85%), independent of whether these animals are still able to display escape behavior in response to visual stimuli (+, responder) or not (-, NR) (Fisher’s exact test, *p* = 0.99). Escape is not evoked by red-light stimulation of control animals with UAS-GFP instead of UAS-CsChrimson expression in LPLC2. **(E** and **F)** Maximum intensity projection views of representative CLSM image stacks of the LC4 interneuron in a >60 days old animal that responded to visual stimuli with escape behavior (responder, E) vs. a >60 days old animal that did not respond to visual stimuli with escape behavior (NR, F) do not reveal massive reductions in LC4 interneuron arbor density or neuropil coverage. **(G)** Optogenetic stimulation of the LC4 interneurons induces escape responses in animals between 3 days and 30 days of age, though with a lower reliability than GF (see Fig 3A) or LPLC2 stimulation (see C). In contrast to optogenetic activation of GF or of LPLC2, escape probability in response to optogenetic activation of LC4 declines significantly during aging (Fisher’s exact test, *p* < 0.0001), it drops markedly in mid-aged (30–50 days) and old flies (50–60 days) and is absent in flies older than 60 days (>60 d). **(H)** Testing the effectiveness of optogenetic stimulation of the LC4 interneurons in animals that are pre-selected to show a behavioral escape response to visual stimulation shows that the effectiveness of LC4 stimulation to elicit escape drops from nearly 100% in young flies to ~50% in aged flies (50–60 days) and is absent in flies older than 60 days. **(I)** Representative images of the LC4 neurons with UAS-GFP expression (green) and selective enlargements of their terminals with UAS-BRP.S (BRP-short, magenta). **(J)** Quantification of BRP.S intensities in the LC4 terminals during aging and in old (>60 days) responders (R) and nonresponders (NR) reveals a slight but not significant increase during aging and a statistically significant decrease in NRs as compared to Rs. **(K)** Cell body count of LC4 neurons remains unaltered during aging and in NRs. **(L)** Representative images of the LPLC2 neurons with UAS-GFP expression (green) and selective enlargements of their terminals with UAS-BRP.S (magenta). **(M)** Quantification of BRP.S intensities in the LPLC2 terminals during aging and in old (>60 days) responders (R) and NR reveals a significant increase during aging but no statistically significant decrease in NRs as compared to Rs. (**N**) Cell body count of LPLC2 neurons remains unaltered during aging and in NRs. See S1 Data for individual data points.

**Fig 8:**
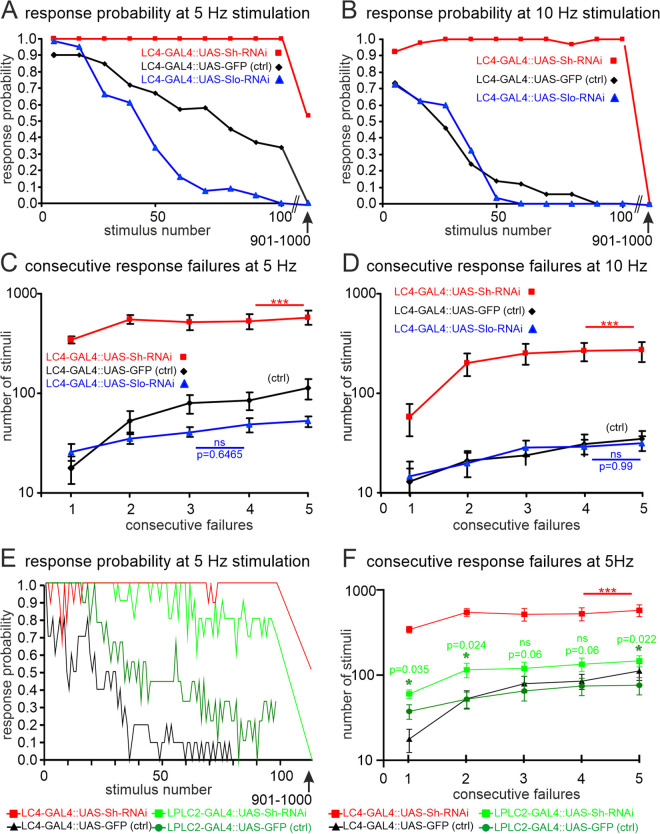
K_v_1 channel knock-down in the LC4 interneuron but not in LPLCs impairs GFS habituation. **(A** and **B)** Habituation of the GF escape circuit is measured as LLR probability in response to 1,000 consecutive electrical stimuli of retina at a frequency of either 5 Hz (A) or 10 Hz **(B)**. Strong frequency dependent decrease in response probability (habituation) is observed within the first 50–100 stimuli in genetic controls (black) and upon RNAi knock-down for the BK channel slo in LC4 visual projection neurons (blue), but no decrease in response probability during the first 100 stimuli occurs upon RNAi knock-down of the K_v_1 channel Shaker in LC4 visual projection neurons (red). **(C** and **D)** A second measure of habituation is the quantification of the number of stimuli (mean ± SEM) that are required before consecutive response failures (from 1 to 5) occur at 5 Hz **(C)** or at 10 Hz stimulation frequency (D). Numbers of stimuli were log transformed (y-axis). In controls (black) and upon RNAi knock-down of slo in LC4 (blue), first response failures are observed after <50 stimuli, and the number of consecutive failures increases up to 5 in a frequency dependent manner within the first 100 stimuli at 5 Hz **(C)** and within the first 50 stimuli at 10 Hz (D). No significant difference is detected (ANOVA) between control and RNAi knock-down of the BK channel slo, neither at 5 Hz (C), nor at 10 Hz (D). By contrast, upon RNAi knock-down of Shaker in LC4 significantly (ANOVA with planned post hoc comparison) more stimuli are required to induce consecutive response failures, both at 5 Hz stimulation **(C)** and at 10 Hz stimulation (D). **(E)** Habituation measured as response probability decrease during 5 Hz stimulation for flies expressing Shaker (Sh) RNAi in LC4 (red) versus in LPLC2 (light green) visual projection neurons plus the respective genetic controls. Both controls and Sh-RNAi in LPLC2 exhibit normal habituation. In contrast, the knock-down of Shaker by RNAi in LC4 neurons causes a failure of habituation within the first 100 stimuli. **(F)** Similarly, upon RNAi knock-down of Shaker in LC4, significantly more consecutive stimuli are required to cause consecutive response failures as compared to Shaker RNAi knock-down in LPLC2 or in genetic controls (ANOVA with planned post hoc comparisons). Although RNAi knock-down of Shaker in LPLC2 affects habituation highly significantly less than RNAi knock-down in LC4, planned comparisons (multiple *t* tests with Welch correction) between LPLC2 with Shaker RNAi and LPLC2-GFP control reveal mild but significant differences. See S1 Data for individual data points.

As mentioned above, in contrast to the loss of LLR in old nonresponding animals, SLR remains intact until the end of life. Our interpretation is that the circuit elements downstream of the GF are likely sufficiently intact to mediate escape behavior in vivo at all ages. This interpretation is further supported by optogenetic stimulation of the GF descending neuron. Following the targeted expression of a red-light shifted channelrhodopsin (UAS-CsChrimson, see Methods) in the GF descending neuron, escape responses can be elicited by a 50 ms red-light flash with ≥80% response probability throughout adult life (Fig 4A, red bars). Controls that express UAS-GFP instead of UAS-CsChrimson under the control of the same GF specific GAL4 driver show no response to the red-light flash (Fig 4A, black bars). Even when testing animals that are older than 60 days and do not show escape behavior to visual stimulation anymore, escape behavior is evoked by optogenetic stimulation of the GF descending neuron in ~50% of the cases (Fig 4B). In sum, these experiments show that, throughout life, optogenetic activation of the escape circuit downstream of the LC4 and LPLC2 interneurons suffices to initiate escape behavior. This is consistent with intact GFS circuitry downstream of the GF.

However, as the number of old NRs in which initiation of escape by optogenetic stimulation of the GF descending neuron does not reach control values, we cannot entirely exclude aging-related functional decline in or downstream of LC4 and LPLC2 like in the thoracic part of the GFS circuitry that is compensated for by alternative circuit routes or impairment of conduction in the GF itself. The GF transmits via mixed electrical/chemical synapses directly to the TTM Mn (see Fig 2A for schematic and Fig 4C for corresponding data from the VNC connectome) but only indirectly via the PSI to the DLM Mns (see Fig 2A for schematic and Fig 4D for corresponding data from the VNC connectome). The extra chemical synapse from the PSI to the DLM Mns (Fig 4D, magenta) adds ~0.5 ms delay [50,51], so that the initiation of the jump precedes that of flight. However, additional outputs from the GF to a total of 157 cell types have been identified in the VNC connectome, and these allow for information transfer from the GF to the DLM Mns via numerous additional thoracic circuit routes [53]. Therefore, even upon functional decline of the PSI, there are additional routes for information transfer from the GF to the DLM Mns. However, neither the short duration of the SLR, nor a constant delay of 0.5 ms between the TTM and the DLM would be in agreement with multiple additional chemical synapses between the GF and the DLM Mns. To further exclude the possibility that GF to DLM Mn circuitry also declines in old NRs but is bypassed by parallel circuit routes, we conducted double recordings of the TTM and the DLM in response to electrical stimulation of the GFS (Fig 4F–4J). First, for both LLR and SLR we observe the reported 0.5 ms delay that is consistent with a direct electrical GF to TTM Mn connection and one, but not more, additional chemical synapse to the DLM Mns (Fig 4F and 4G). Second, this TTM-DLM delay remains similar in >60 days old responders and age-matched NRs (Fig 4I), which is not in line with additional polysynaptic connections between the GF and the DLM Mns as bypass routes. However, there is also the possibility of an additional monosynaptic route between the GF and the DLM Mns that could in principle bypass the PSI without adding additional time to the TTM Mn-DLM Mn delay. The GF reportedly also makes electrical synapses to the so-called GFC1–4 neurons [54]. Further analyses of the VNC connectome reveals that the GFC2 indeed makes chemical monosynaptic connections to the DLM Mns (Fig 4E, GFC2 to DLM Mn synapses in magenta). To test whether this provides a functionally reliable bypass route that connects the GF to the DLM Mns with only one chemical synapse, we conducted double recordings of the TTM and the DLM in animals with genetically ablated PSI (by selective expression of UAS-hid in the PSI). Without the PSI, extracellular electrical stimulation of the GF always induced a short latency response (SLR) in the TTM (Fig 4H, top trace) with an SLR of below 1 ms. This is consistent with a direct electrical-chemical connection of the GF to the TTM and previous reports [50,51,59]. By contrast, the SLR response as read-out from the DLM is absent in 7 of 10 animals tested (Fig 4H, bottom trace). This is not due to technical issues, nonfunctional DLM Mns or neuromuscular defects, because DLM responses can be observed spontaneously or in response to wind stimuli to the head in the same recordings that reveal a lack of SLR in the absence of PSI (Fig 4G). To test whether animals that do not display SLR in the DLM and to test how reliable UAS-hid expression indeed ablates the PSI, we combined SLR measurement in animals that express UAS-hid in the PSI with visualization of PSI ”y co-expression of UAS-tdTomato (for technical reasons only on the right side). Expression of UAS-hid was confirmed by co-expression of UAS-stinger on the same chromosome, resulting in a GFP signal in the nucleus if expressed. We found that in 100% of the cases in which no SLR could be elicited in the DLM but in the TTM, both PSI neurons were ablated (Fig 4H, left). Vice versa, animals that showed SLR in both DLM and TTM had both PSI neurons in 100% of the cases (see also S1 Fig). However, in ~75% of the animals that expressed UAS-hid in the PSI (expression of UAS-transgenes was confirmed by co-expression of UAS-tdTomato and UAS-stinger), the PSI neurons were indeed gone (Fig 4H, right). This strongly supports the GF – PSI – DLM Mn route as the route by which visually induced escape that runs via the GF descending neuron is transmitted to induce flight and hints that an alternative route via GFC2 neurons is unlikely.

To test effectiveness of neuron ablation by expression of UAS-hid, we also tested other Split-GAL4 drivers. Expression of UAS-hid UAS-stinger leaves one GF in 50% of the animals and both in the other 50% of the animals. In LC4 neurons, ~99% of the neurons are ablated upon UAS-hid UAS-stinger expression. By contrast, expression of UAS-hid in DLM Mns ablates these neurons faithfully (S1 Fig).

These data support the interpretation that the circuit elements downstream of the GF are robust to aging-related functional decline, although the possibility of some decline remains because escape can only be elicited in 50% old NRs if the GF is optogenetically stimulated (Fig 4B). For the TTM and the DLM Mns this is further supported by our finding that independent of age and responsiveness, SLR is always observed, although there is a slight increase in SLR delay by ~0.2 ms for both the TTM (Fig 4J) and the DLM read-outs (Fig 2C).

### Localizing the neural correlates for acute stress related loss of escape behavior

Does the differential vulnerability of the escape circuit that we observe during aging occur also in response to acute stressors, such as food deprivation (starvation)? To test this, 39 young flies (5 days old) are transferred to individual food vials and the functional status of each fly is examined every 3 hours until death (Fig 5A). The first flies die after 12 hours of starvation, and no fly survives starvation for more than 74 hours (Fig 5A). However, roughly 75% of the flies (28/38) show no obvious physical impairment, keep their ability to climb the wall of the vial, and show escape responses until at least three hours before death (last assessment before death), but the flies show increased locomotor activity. We term flies that show escape responses and no obvious impairments starved responders (SRs, Fig 5A, gray bars). By contrast, longitudinal testing also identifies starved flies that exhibit climbing response failures and other motor disabilities, similar to those observed in aged animals prior to death [25]. During their late-life impairment span these flies do not show escape responses anymore (Fig 5A, starved NRs, SNRs). Selecting flies from longitudinal starvation studies for electrophysiological testing of the escape circuit shows that in starved responders, electrical stimulation of retina reliably evokes LLR responses in the DLM with the typical delay of slightly above 4 ms (Fig 5B and 5C, black). By contrast, LLR is consistently absent in starved NRs (Fig 4B and 4C, red). These data correspond to our findings during aging, where old responders show LLR but old NRs do not (Fig 2I and 2J). Moreover, also in correspondence to what we observe during aging, direct stimulation of the GF descending neuron reliably induces a SLR both in starved responders and in starved NRs (Fig 5D and 5E). However, in starved NRs SLR is slightly (roughly 0.2 ms) but significantly increased, as has previously also been reported for aging [52]. Finally, upon starvation neither refractory period nor following frequency 50 is different between responders and NRs (Fig 5F and 5G), which is also similar to aging (Fig 2E–2G). Together, these data indicate that the GF descending neuron and downstream escape circuit elements are highly resilient to aging and acute starvation induced stress, but circuit elements responsible for LLR are vulnerable. Importantly, starvation induced circuit failure can be rescued by re-feeding. In fact, although LLR is absent in starved NRs, it is regained within 1 hour of feeding in the same animals (Fig 5B and 5C). Interestingly, upon re-feeding LLR becomes even faster as compared to before starvation induced failure (Fig 5B and 5C). Similarly, the short latency responses as induced by GF stimulation (SLR), which are slightly but significantly increased in starved responders, becomes significantly faster through re-feeding (Fig 5D and 5E). This indicates that starvation induced failures of LLR, or increases in SLR are caused by energy shortage that can be quickly recovered by energy re-supply.

We next tested whether LLR is also selectively affected by exposure to other acute stressors. Exposing young flies at the age of 5 days to oxidative stress (see Methods) also eliminates LLR without affecting SLR (see S2 Fig). Separating responding from nonresponding flies after the continued exposure to oxidative stress for 2 days shows that SLR remains intact in all animals, responders and NRs. By contrast, in all NRs LLR is absent, whereas it is reliably present in age-matched flies that remain responsive after having been exposed to oxidative stress for the same duration as the NRs (S2 Fig, left). For SLR neither the duration, nor the refractory period, or the following frequency 50 are affected in NRs as compared to responders. And finally, we also observe the selective loss of LLR but not SLR in animals that lose their responsiveness during desiccation (S2 Fig). Therefore, information transfer from the GF to the DLM muscle is resilient to aging, desiccation, oxidative stress and starvation induced loss of escape responsiveness. However, the circuit elements vulnerable to aging, acute energy shortage, acute desiccation, or acute oxidative stress must be upstream of spike initiation in the GF descending neuron. But what are the vulnerable circuit elements?

### GF dendritic structure changes correlate with aging but not with functional decline

The next step upstream of GF spike initiation is synaptic input to GF descending neuron dendrites. The GF has three primary dendrites in the central brain, only one of which receives visual input (Fig 6A, red). Visual information relevant to escape is relayed from the optic lobes to the GF visual dendrite by two groups of visual projection neurons, LC4 and LPLC2 neurons (see schematic in Figs 2A and 3). Synaptic inputs of LC4 and LPLC2 to the GF dendrites have been quantified from adult *Drosophila* whole-brain electron microscopy volumes [55,44]. LC4 and LPLC2 neurons together make >98.5% of the inputs from optic lobe to the GF visual dendrite. Therefore, nearly all visual information relevant to escape converges via LC4 and LPLC2 synapses onto the GF dendrites. Geometric reconstructions of GF dendrites from confocal image stacks [60,61] reveal some age-related structural differences between young and old animals (Fig 6B and 6D). Although overall dendritic structure appears similar in weeks 3 and 5 of adult life (Fig 6B), and quantification of total dendritic length reveals no differences between young (1–3 weeks), middle aged (5–7 weeks) and old animals (9–11 weeks, Fig 6D, top), the number of dendritic branches is significantly smaller in middle aged (5–7 weeks) as compared to young (1–3 weeks) animals, but then remains stable until close to the end of life (Fig 6D, bottom). The analysis shown refers to the entire GF dendrite, but it also holds for the visual dendrite only.

It seems unlikely that the loss of dendritic branches is causative to the loss of behavior, because the structural changes occur earlier than behavioral defects (Fig 1B). This interpretation is further confirmed by relating the quantification of GF dendritic structure not only to chronological aging but also to functional aging (Fig 6C and 6E). First, side-by-side comparisons of reconstructions from responders and NRs at 7, 9, and 11 weeks of age reveal no obvious differences (Fig 6C). Second, quantification of dendritic structure in old responders and old NRs reveals no significant differences in total dendritic length or the number of branches (Fig 6E). And third, the Dα7 nAChR, which is known to be the postsynaptic receptor for visual input to the GF dendrite and required for escape behavior [62], is expressed at comparable levels and densities in the GF dendritic space, no matter whether old animals show escape behavior (responder, Fig 6F, bottom) or not (NR, Fig 6F, top). To test whether the postsynaptic site of cholinergic synapses may be impaired on the level of protein expression, we quantified the expression levels of the postsynaptic cholinergic synapse marker Drep2 [63] selectively in the GF descending neuron and assessed the number of Drep2 puncta in the GF dendrites that receive input from the LPLC2 and LC4 visual projection interneurons. This reveals no obvious differences in GF visual dendrites between >60 days old nonresponding and age-matched responding animals (Fig 6G, see also S3 Fig for a side-by-side comparison of the GF dendrites with Drep2 expression in 5 responders and 5 NRs). Quantification of the number of postsynaptic Drep2 puncta in the GF dendrite confirmed that there are no statistically significant differences between old NRs or old responders as well as middle aged responders (Fig 6H). In sum, GF dendritic structure exhibits some decrease in the number of dendritic branches with age, but this does not account for the aging-related loss of escape behavior. Similarly, there are no indications for a reduction in the numbers of postsynaptic nAChR expression in the GF. Therefore, aging-related circuit decline likely occurs upstream of the GF descending neuron.

### Aging affects information transfer from LC4 but not LPLC2 interneurons to the GF

The LPLC2 and the LC4 visual projection interneurons relay information from the optic lobe to the GF descending neuron (see above and Fig 3). We combine structural analyses with optogenetics to probe for potential aging-related defects in these visual projection neurons. Utilizing highly selective split-GAL4 fly lines allows for targeted expression of UAS-GFP as reporter or UAS-CsChrimson for optogenetic activation in either the LPLC2 or the LC4 neurons (Fig 7). Comparing the overall neuropil coverage of LPLC2 projection neuron dendrites in old responding (Fig 7A) and old nonresponding (Fig 7B) animals reveals no obvious differences with respect to neuropil coverage, dendritic density, and the coverage of their axon terminals (see also S4 Fig for side-by-side comparisons of LPLC2 neurons and their BRP marked axon terminals in old responders and NRs). However, functionally LPLC2 neurons remain intact throughout adult life, because optogenetic activation of animals expressing CsChrimson selectively in LPLC2 neurons reliably elicits escape responses in >80% of all animals tested, independent of age (Fig 7C). Moreover, optogenetic activation of LPLC2 elicits escape with similar reliability in old animals that do not show escape behavior in response to visual stimulation (Fig 7D). Therefore, escape responses can be induced by LPLC2 activation in animals that have lost their normal behavioral response during aging. Controls without UAS-CsChrimson expression in LPLC2 do not respond to the red-light flash (Fig 7D). Therefore, LPLC2 is unlikely causative to the aging-related decline in escape responsiveness.

Repeating the same experiments with LC4 projection neurons yields different results. Although the LC4 projection neuron morphologies show no obvious differences when comparing old responding (Fig 7E) and old nonresponding (Fig 7F) animals, optogenetic activation of LC4 neurons fails to elicit escape in old animals (Fig 7G and 7H). Following the expression of UAS-CsChrimson under the control of an LC4 projection neuron specific split-GAL4 driver line, red-light flashes induce escape in ~25%–30% of all animals in the age groups of 3–10 days and 10–30 days (Fig 7G). Therefore, optogenetic stimulation of LC4 induces escape in young animals, but with an approximately 3-fold lower likelihood as compared to optogenetic stimulation of LPLC2. By contrast, the escape response probability upon LPLC2 optogenetic stimulation does not decrease with increased age (Fig 7C), whereas the response probability upon LC4 optogenetic stimulation decreases drastically with age (Fig 7G). The initial response likelihood of 25–30% in young animals drops ~3-fold to <10% in the age group of 30–50 days, another 3-fold to <3% in the age group of 50–60 days, and it is nearly zero in animals older than 60 days (Fig 7G). This age-related decline of information transfer from LC4 projection neurons to the downstream elements of the escape circuit is also evident when testing only animals that still show visually induced escape behavior. Upon pre-selection of responding animals by behavioral testing, optogenetic activation of LC4 induces escape responses with near 100% reliability in young (10–30 days) and mid-aged (30–50 days, Fig 7H) flies. By contrast, response probability drops ~2-fold to 50% in the age group 50–60 days, and is nearly zero in animals older than 60 days (Fig 7H). Therefore, information transfer from LC4 to the downstream circuit is severely impaired in old animals, even if these still show escape behavior as mediated by the activation of LPLC2. These data indicate that synaptic transmission from LC4 projection neurons to the GF descending neuron may be the site for aging-related escape motor behavioral decline. It seems unlikely that the defect is postsynaptic in the GF dendrites, because even in old nonresponding animals these remain structurally intact (Fig 6F), express normal amounts of nAChRs (Fig 6F–6H), and respond to LPLC2 stimulation (Fig 7D). What makes the LC4 to GF synapse more vulnerable than other synapses in the circuit, in particular the LPLC2 projection neuron to GF synapse?

To pinpoint factors that could underlie functional decline of information transfer from the LC4 visual projection neurons to the GF descending neuron, we first tested for a potential loss of visual projection neurons at old ages. We quantified the numbers of LC4 neuron cell bodies in young (3 days old), mid-aged (40 days old) as well as old (>60 days old) responding and age-matched nonresponding animals (Fig 7K), and for comparison we conducted the same analysis with LPLC2 neurons (Fig 7N). Our cell body counts match those from the hemibrain v1.2.1 connectomics data set ([44]; ~70 LC4 neurons and ~85 LPLC2 neurons), but are slightly lower as compared to previous reports [33] that are based on another adult *Drosophila* whole-brain electron microscopy volume [55]. However, we find no changes in cell body numbers through aging or in old NRs, neither with respect to LC4 neurons (Fig 7K), nor with respect to LPLC2 neurons (Fig 7N).

Next, we estimated the number of presynaptic sites on LC4 as well as on LPLC2 axon terminals that connect to the GF dendrites. We expressed the strawberry-tagged active zone marker BRP-short (BRP.S, [64]) under the control of LC4-Split-GAL4 (Fig 7J) or LCLP2-Split-GAL4 (Fig 7M) and quantified the total fluorescence intensity that resulted from strawberry-tagged BRP.S puncta in the neuropil volume where these visual projection neurons synapse onto the GF dendrite. Comparing LC4 and LPLC2 in 3–10 days and 30–50 days old animals as well as in >60 days old responders and age-matched >60 days old NRs (Fig 7J and 7M; see also S4 and S5 Figs) yields two observations. First, according to previously published data on other *Drosophila* central synapses [14], we find a gradual increase of BRP expression in axon terminals between the ages of 10 and 60 days in both LC4 (Fig 7J) and LPLC2 neurons (Fig 7M). Aging-related increases in expression levels of the active zone protein BRP have been referred to as BRP ramp-up and are associated with functional changes ([14,27] see discussion). Second, the loss of LC4 to GF information transfer goes along with a significant decrease in BRP.S intensity in LC4 presynaptic terminals of old NRs as compared to age-matched responding animals (Fig 7J). By contrast, LPLC2 presynaptic terminals also show BRP.S ramp-up through aging but no decrease in BRP.S in old NRs (Fig 7M). These data show that the functional decline of LC4 to GF information transfer is accompanied by the selective decrease of presynaptic active zone protein expression in LC4 neurons.

### LC4 visual projection neurons are major players in escape circuit plasticity

Given that the above data indicate that LC4 to GF information transfer undergoes functional decline in old NRs, and that this decline goes along with reduced expression of BRP in presynaptic active zones, we next tested whether the LC4 to GF synapse displays different characteristics than the LPLC2 to GF synapse. The giant fiber system (GFS) mediated visually induced escape response shows habituation [65], one of the simplest forms of learning. Habituation is defined as a reduction in response likelihood to the repeated presentation of a stimulus that is not attributable to sensory adaptation or motor fatigue [66]. The GFS response is all-or-none, and the likelihood of an LLR response decreases with repeated stimulation, but full response likelihood can be restored (dishabituation) by a novel stimulus, such as an air puff to the head [57]. Moreover, GFS habituation is affected by mutations in potassium channels [58]. For example, habituation is enhanced (premature habituation) in mutants for the *Drosophila* K_v_1 channel Shaker (Sh), while it is reduced in mutants for the *Drosophila* BK channel slowpoke (slo) (delayed habituation; [57,58]). However, in mutants the cellular locus of the effect remains unknown because all neurons carry the gene defect for the respective channel. To pinpoint the cellular site of habituation, we manipulate the expression of specific potassium channels in selected neurons of the escape circuit by the targeted expression of respective UAS-RNAi transgenes under the control of highly specific split-Gal4 drivers to then test the resulting effects on GFS habituation upon electrical stimulation of retina. These stimulation and read-out protocols to probe for GFS habituation have first been established by [57,58]). In controls, stimulation of retina at either 5 Hz (Fig 8A) or at 10 Hz (Fig 8B) reveals a clear, frequency dependent decline in response probability. Following targeted RNAi knock-down of the BK channel slo in the LC4 visual projection neurons only slightly accelerates habituation at 5 Hz stimulation frequency (Fig 8A, blue line) and does not affect habituation at 10 Hz stimulation. A consistent quantitative measure of the level of habituation is provided by the number of stimuli after which 1–5 consecutive response failures occur [57]. There are no significant differences between control and slo-RNAi in LC4 with regard to the number of stimuli after which 1, 2, 3, 4, or 5 consecutive response failures occur, neither for 5 Hz stimulation (Fig 8C) nor for 10 Hz stimulation (Fig 8D). By contrast, targeted RNAi knock-down of the K_v_1 homolog, Shaker, selectively in the in LC4 visual projection neurons abolished normal habituation. Both at 5 Hz (Fig 8A) and at 10 Hz stimulation (Fig 8B) no decrease in response likelihood is observed during the first 100 stimuli (red lines). In controls, after 100 stimuli response probability is down to 35% at 5 Hz stimulation and down to 0% at 10 Hz stimulation (Fig 8A and 8B, black lines). Similarly, the number of stimuli needed to induce consecutive failures is highly significantly increased upon Shaker RNAi knock-down in LC4 (Fig 8C and 8D, red lines) as compared to control (Fig 8C and 8D, black lines) or slo-RNAi (Fig 8C and 8D, blue lines). Importantly, this effect is specific to LC4 visual projection neurons. Expression of the same Shaker RNAi transgene in LPLC2, the other visual projection neuron type presynaptic to the GF descending neuron, has a minor effect on response probability (Fig 8E) and the number of consecutive failures upon repeated stimulation (Fig 8F). Therefore, habituation in the GF escape circuit requires the Shaker K_v_1 potassium channel in the LC4 visual projection neurons. We conclude that LC4 interneurons are major players in GFS habituation, and note these LC4s are the neurons that fail to transmit the signal to the GF late in life as well as upon exposure to different acute stressors.

## Discussion

Mapping the age-related loss of *Drosophila* escape motor behavior into the brain shows that many circuit components are robust against aging and retain function even in old flies that have lost the behavior. By contrast, age-related behavioral decline is caused by the decay of one synapse type in the central brain, which is a major plastic element in the escape circuit. This makes us suggest that a trade-off for plasticity might be vulnerability to aging.

### Hard wired sensory and motor components of the escape circuit are robust to aging

Our results suggest that neither phototransduction nor synaptic transmission from photoreceptors to first order interneurons is impaired by aging, because ERGs reveal no significant differences between young and old animals, or animals that have lost their escape response. Although these experiments do not rule out subtle changes in photoreceptor or lamina interneuron physiology, we conclude that such changes do not underlie the loss of visually induced escape in old animals. Similarly, the components mediating the last few steps of information flow from the CNS to muscle are surprisingly resilient to aging. First, electrical stimulation of the flight as well as the jump motoneurons is reliably transmitted to the DLM and the TTM muscle fibers in young and old responding and age-matched nonresponding animals. Although aging-related decline of various motor behaviors has been reported in *Drosophila* [23,25,26] this has not been pinpointed to functional decline of motoneurons. Second, the SLRs that are evoked in the DLM and the TTM fibers upon electrical stimulation of the GF descending neuron remain present even in old NRs. SLR in the TTM fibers is a measure for action potential propagation along the GF axon as well as signal transmission from the GF to the TTM motoneuron and then to the TTM. Although SLR in the TTM is increased by roughly 0.2 ms in old NRs, transmission between the GF and the TTM remains faithful even in NRs. The small (~0.2 ms) but statistically significant increase in transmission between the GF and the DLM (SLR) in old flies is consistent with a previous report and has been attributed to aging-related changes in insulin signaling [52]. However, this cannot explain the loss of escape behavior in old flies because all flies tested showed SLR, even NRs. SLR in the DLM fibers is a measure for faithful action potential propagation along the GF axon as well as signal transmission from the GF to the DLM Mns and neuromuscular transmission to the DLM fibers. The GF connects to the DLM Mns via the peripherally synapsing interneuron (PSI), and genetic removal of PSI eliminates SLR in the DLM in 80% of the cases. In the remaining 20% SLR remains with unchanged latency upon PSI removal. A likely explanation is a parallel GF to DLM Mns route. It has been reported that the GF also makes electrical synapses onto the GFC1–4 neurons [54], and our analysis of the VNC connectome [53] reveals that the GFC2 neurons in fact synapse onto the DLM Mns. This can explain why SLR remains in some animals upon PSI removal, and thus adds uncertainty to the idea that PSI is robust against aging. However, given that all old NRs show SLR, but only 20% of the animals without PSI show SLR, the most parsimonious interpretation is that PSI is also largely robust against aging. We therefore suggest that all motor circuit components between the GF and the muscles are highly robust against aging-induced functional decline, as has also been suggested by Iyengar and colleagues [67].

### Aging-related dendritic structure changes do not necessarily impair function

Small, region- or neuron type-specific changes in dendritic structure and spine density are characteristic of the effects of brain aging on neuronal morphology [11], but it is not always clear whether these structural alterations are causative to functional decline. Our data demonstrate functional circuit decline upstream of GF spike initiation, and in fact, we find significant age-related decreases in GF dendritic branch numbers. However, these are not the cause of functional decline. First, GF dendritic branch numbers are decreased already after 5–7 weeks of adult life, before behavioral decline is observed (after 7–9 weeks of age). Second, there are no differences in GF dendritic branch numbers between old responding animals and age-matched nonresponding animals. Therefore, in the case of the GF descending neuron, age-related decreases in dendritic branch numbers do not correspond with the loss of responsiveness. Third, optogenetic stimulation of the LPLC2 visual projection neuron that connects to the GF dendrites reliably evokes escape response across all age groups tested, even in ~9-week-old, nonresponding animals. Therefore, even in old animals that have lost their behavioral responsiveness and roughly 35% of the GF dendritic branches, excitatory synaptic input to the GF dendrites reliably evokes escape responses. This corresponds to previous findings in *Drosophila* motoneurons that retain basic function even upon the loss of 90% of their dendrites [68]. Together, our results rule out a contribution of dendritic branch loss to functional decline in this system. This underscores the importance to experimentally explore the functional consequences of the diverse changes in dendrite structure that are observed across species in a neuron-type specific manner. In rats, hippocampus aging does not affect granule cells dendrite shape [69], whereas CA1 pyramidal neurons exhibit increased dendrite length and distal branching [70]. Typically, reductions rather than increases in dendrite complexity are reported, as, e.g., for rat prefrontal cortex pyramidal neurons [71] and mouse neocortical pyramidal neurons [7]. For the latter, functional data suggest that decreased dendritic structural plasticity contributes to reduced functional plasticity during aging. However, functional interpretations based on aging-related dendritic structure changes can be misleading and require careful experimental validation in each species and type of neuron investigated.

### Only distinct subsets of synapses are vulnerable to aging and acute stressors

Our data suggest information transfer between the LC4 visual projection neurons and the GF descending neuron to be vulnerable not only to aging but also to acute starvation induced or oxidative stress. First, electrophysiological characterization of the escape circuit restricts the site of functional decline to circuit components upstream of the GF, because NRs lack LLR but exhibit SLR. Second, optogenetic GF stimulation reliably evokes escape (in >80% of the animals) at ages when visual stimulation fails to induce escape in >90% of the animals. Therefore, with some uncertainty about the PSI neuron (see above) the circuit components downstream of GF spike initiation function even in old NRs. Interestingly, selective LLR failure with SLR still being reliably observed also occurs in response to other stressors, such as starvation induced energy shortage (see Fig 5) or oxidative stress (see S2 Fig). This indicates that the same circuit elements are vulnerable to aging and to other pathology inducing stressors, or that normal aging renders these elements more susceptible to additional pathological challenges. In Alzheimer’s disease (AD) research, the phenomenon that brain region-specific vulnerability to aging renders these regions more susceptible to pathological changes has been referred to as ‘normalcy-pathology homology’ [1].

Taken together with our findings that phototransduction and synaptic transmission from photoreceptors to first order visual interneurons function throughout adult life, even in old NRs, our data restrict the site of circuit vulnerability to a locus downstream of lamina interneurons and upstream of the GF. The visual projection neurons LPLC2 and LC4 make up 98.5% of the direct input synapses that the GF receives from the optic lobe [33,44,55]. The LPLC2 to GF synapse can be ruled out as aging-sensitive circuit element, because optogenetic stimulation of LPLC2 reliably induces escape (in ≥80% of the animals) in old animals with a confirmed loss of escape behavior. By contrast, optogenetic stimulation of LC4 induces escape responses with a lower probability as compared to LPLC2 or GF stimulation, even in young animals. This could be due to a weaker synaptic connection of LC4 to the GF as compared to LPLC2 to GF, or alternatively, it could be due to lower UAS-CsChrimson expression levels in LC4 as compared to LPLC2, or both. However, we confirmed with UAS-GFP that the LC4 and the LPLC2 split GAL4 lines both drive the expression of UAS-transgenes through all ages, even in old NRs. Moreover, when selecting only animals that display visually induced escape behavior, optogenetic stimulation of LC4 induces escape with nearly 100% reliability up to the age of 50 days. This matches the age before behavioral decline as observed in wildtype flies. Moreover, in animals 50–60 days old, escape response likelihood is reduced to 50%–60% for both visual stimulation in wildtype flies and optogenetic stimulation of LC4 in responders. Finally, animals older than 60 days rarely respond to visual stimulation and almost never to optogenetic stimulation of LC4. These data suggest that information transfer from LC4 to GF is vulnerable to aging. This is in agreement with structural changes that we observe selectively at the LC4 presynaptic terminals that target the GF. On the one hand both types of visual projection neurons, LC4 displays a trend and LPLC2 displays an aging-related increase in the expression of the presynaptic active zone marker BRP. This has also been reported for other aging neuron types in the *Drosophila* [14] and the honeybee [72] brain. In *Drosophila*, this has been referred to as BRP ramp-up, been associated with age-related memory defects and mechanistically linked to autophagy defects [14,27]. Therefore, both LC4 and LPLC2 neurons display presynaptic changes that are known in aging neurons. On the other hand, only LC4, but not LPLC2 neurons, show a significant decrease in BRP expression at presynaptic active zones that occurs selectively in old NRs but not in age-matched responders. This is consistent with the interpretation that a decline in LC4 presynaptic function underlies the behavioral loss of the escape response. Although we cannot exclude additional aging-related defects in visual interneurons between lamina and lobula [73,74], the transmission failure from LC4 to GF that we observe in old flies is sufficient to explain the aging-related behavioral decline in escape responsiveness.

### The plastic circuit component is vulnerable to aging

Importantly, the LC4 to GF synapse is not only the circuit part vulnerable to aging, it is also the one circuit component critical for a simple form of learning, habituation. Although, it has previously been shown that mutations for different voltage and calcium gated potassium channels affect GFS habituation in various ways [57,58,65], the site of circuit habituation remained unknown. We now demonstrate that the selective knock-down of the K_v_1 homolog Shaker in LC4 visual projection neurons eliminates GFS habituation, whereas the same knock-down expressed in LPLC2 visual projection neurons has only a minor effect. Therefore, LC4 to GF synapses are the main plastic ones in the circuit and are most vulnerable to aging. Similarly, in the mammalian brain it has been suggested that brain areas characterized by high degree of plasticity are those most vulnerable to detrimental effects of aging, and moreover, that this vulnerability to aging renders them also more susceptible to neurodegenerative diseases [1,75,76] and potentially other stressors. Similarly, we find the circuit elements susceptible to aging also most vulnerable to different acute stressors. We do not claim that aging, starvation, and oxidative stress all converge on the same cellular and molecular causes for functional decline, especially not because aging and oxidative stress induced failure are irreversible, whereas starvation induced failure is reversible. In the light that we identify the LC4 to GF synapse tunable to undergo habituation in response to repeated stimulation, we speculate that it must thus not operate with a safety plateau that ensures information transfer even upon reductions in function. This might be the reason why the plastic synapse is the first to fail upon exposure to the various function reducing stressors that we have tested, including aging, starvation, and oxidative stress.

In neocortical circuits and in hippocampus plasticity is characterized by the remodeling (expansion, retraction, and stabilization) of a class of dendritic postsynaptic specializations, dendritic spines [77]. In rodents, dendritic spine plasticity is related to long-term memory [78], and spine density in prefrontal cortex is reduced during aging [79]. Thin spine plasticity has been connected to long-term memory and memory decay during aging to a decrease in the density of thin spines in rodents [80–83], nonhuman primates [84], and humans [85]. In general, in the mammalian brain the prefrontal cortex and the hippocampus represent highly plastic brain parts that are particularly vulnerable to aging. Therefore, aging often manifests in alterations of LTP and long-term memory, defects in the dynamics of place cells and spatial memory, as well as decreased associative and working memory abilities [11].

Although with caution, it has been suggested that brain regions characterized by life-long plasticity might be particularly susceptible to aging. Our data support this view and extend it beyond the mammalian brain by showing that the main plastic element of that *Drosophila* escape circuit is susceptible to aging and other stressors, whereas the hard-wired components are highly robust. Thus, we suggest that the price for brain plasticity and behavioral adaptability may be increased vulnerability. On the other hand, solid evidence shows that neuroplasticity plays crucial roles in maintaining and restoring brain function upon injury, neurodegeneration, and aging [86]. Therefore, neural plasticity seems like the Janus head of the aging brain, contributing to both cause and relief of functional decline.

## Methods

### Animals

#### Fly rearing.

Flies were kept at 25 ± 1 °C and 60 ± 5% relative humidity and a 12 hours light/dark regime in 25 mm diameter plastic vials with mite proof foam stoppers on a standard cornmeal, yeast, agar, glucose diet containing 0.75% (w/v) agar, 4.5% (w/v) dry yeast, 3.5% (w/v) corn meal, 5.5% (w/v) Sucrose, 0.4% (v/v) Propionic acid, and 2.5% (v/v) nipagen diluted in 10% absolute ethanol. If not noted otherwise, from the first day after adult eclosion on, flies were kept in cohorts of 10 animals (5 males and 5 females) in individual food vials. They were transferred to a fresh food vial once per week. For all experiments male flies were removed at the ages required for experimental testing. For starvation experiments male flies were singled out at day 5 of adult life and subjected to starvation in agar vials.

#### *Drosophila* strains.

As wildtype control, we used mainly Oregon-R (BDSC, #5), except that we used CantonS for the video shown in Fig 1A. For optogenetic stimulation, we used highly specific split-GAL4 diver lines to drive the expression of UAS-CsChrimson (BDSC, #55,136) either in the GF descending neuron ([17A04_p65ADZp (attp40); 68A06_ZpGdbd (attP2)], [36]), in the LC4 ([R47H03_p65ADZp (attP40); JRC_SS00315 R72E01_ZpGdbd (attP2)], [87]), or in the LPLC2 ([R19G02_p65ADZp (attP40); R75G12_ZpGdbd (attP2)], [87]) interneurons. As controls we expressed UAS-GFP instead of UAS-CsChrimson under the control of the respective split-GAL4 drivers. UAS-cd4-td-GFP was used to assess the structure of the GF dendrites or the LPLC2 and the LC4 visual projection neurons at different ages. UAS-BRP.S^mstrawberry^ or UAS-Drep2^mstrawberry^ were used to label either presynaptic active zones [64] in LC4 and LPLC2 interneurons or the sites of postsynaptic nAChRs [63] in the GF descending neuron.

### Immunohistochemistry

For dissection of the CNS, animals were cold anaesthetized on ice. Legs and wings were removed, the animals pinned ventral side down with fine minuten pins in a sylgard dish and submerged in dissection saline (composition in [mM]: NaCl 128, KCl 2, CaCl_2_ 1.8, MgCl_2_ 4, HEPES 5, Sucrose ~35.5. Osmolality was adjusted to 300 mOsM/kg with sucrose. pH was adjusted to 7.24 with 1N NaOH). Next, the dorsal thorax and head capsule were cut open along the midline and the animal was carefully bent open by pulling the thoracic cuticle and DLM muscles laterally. Next, heart, and fat tissue were removed to expose the CNS and the preparation was fixated for 1 hour in 4% paraformaldehyde in phosphate buffered saline PBS (0.1 M) for 50 min.

Fixation was followed by 5x rinsing and 6x 20 min washing in PBS (0.1 M). Next, specimens were washed 3x 20 min in PBS-TritonX (0.5%) and incubated with primary antibody in PBS-TritonX-100 (PBS-Tx, 0.3%) over night at 4 °C on a shaker. Primary antibodies were rabbit α-GFP (1:1000, Invitrogen A11122) and mouse α-BRP (1:200, DSHB nc-82). Following incubation with primary ABs, specimens were washed 6x 30 min in PBS and incubated in secondary ABs (donkey α-rabbit-Alexa-488 and donkey anti mouse-Alexa 647, both Invitrogen) overnight at 4 °C on a shaker in the dark. Next, preparations were washed 6x 30 min in PBS, dehydrated in an ascending ethanol series (50, 70, 90, and 100%, 10 min each), cleared and mounted in methylsalicylate. For immunohistochemistry with rat α-Dα7nAChR antibody (kind gift of Dr. H Bellen, BCM, Houston, Texas) we used a different protocol that requires fixation in 4% PFA for 25 min only and has previously been described [88]. For immunohistochemical analysis of BRP intensity in LC4 and LPLC2 presynaptic terminals, UAS-BRP.S^mstrawberry^ [64] and UAS-cd4-td-GFP were co-expressed under the control of Split-GAL4 in either LC4 or LPLC2. Immunohistochemistry was as above except that we fixed for 1 hour in 2% paraformaldehyde in 0.1M PBS (PBS) at room temperature without shaking, washed in 2% PBS-Tx instead of 0.5%, and that primary AB incubation was for 48 hours. Primary antibodies were polyclonal rabbit α-GFP, 1:1000, Invitrogen A11122, monoclonal rat α-mCherry, 1:2500, clone 16D7, Invitrogen M11217 and secondary antibodies were donkey α-rabbit Alexa 488, Thermo Fisher A21206, donkey α-rat Alexa 647, Jackson Immunoresearch 712-605-150, both 1:500 in 0.1% PBS-Tx. For the analysis of Drep2 puncta in GF visual dendrites, we expressed UAS-Drep2^mstrawberry^ and UAS-cd4-td-GFP under the control of GAL4 in the GF. Primary antibodies were polyclonal chicken α-GFP, 1:1000 (Invitrogen A10262) and monoclonal rat α-mCherry, 1:2500, clone 16D7 (Invitrogen M11217) in 0.5% PBS-Tx. Secondary antibodies were donkey α-chicken Alexa 488, Jackson Immunoresearch 703-545-155, and donkey α-rat Alexa 594 (Thermo Fisher A21209) both 1:500 in 0.1% PBS-Tx.

### Confocal laser scanning microscopy (CLSM) and 3-D neuronal geometry analyses

Except for imaging Drep2 puncta in GF dendrites all images were acquired with a Leica SP8 confocal laser scanning microscope (Leica Microsystems Inc., RRID:SSR_004098) with excitation wavelengths at 488 nm (Argon laser) and/or at 633 nm (HeNe laser). Detection was conducted with photomultipliers at wavelengths between 495 and 540 nm and between 640 and 690 nm, respectively. Overview images were acquired at a xy pixel resolution of 0.189 × 0.189 µm and z-step distances of 1 µm. High resolution images for analyses of dendrites visual projection neuron axon terminals were acquired with a 40x oil lens (N/A 1.3) at a pixel resolution of 83 × 83 nm and a z-step distance of 300 nm. All images were stored as Leica lif. files and further processed with LasX (Leica Microsystems), ImageJ software (both freeware), or with Amira 5.3.3 (Thermo Scientific) and custom written 3-D neuronal geometry reconstruction plugins [60,61]. For quantitative comparisons of UAS-BRP.S expression levels, all specimens were treated in the same histology and immunocytochemistry run, and image acquisition was done in the same imaging session with identical laser and detector setting. Image analysis was conducted with Fiji open-source software. For analysis of fluorescence intensity of BRP.S in LC4 and LPLC2 terminals the structure was first viewed as maximum projection view, encircled and then all structures outside the marked area were removed. Then fluorescence intensity of each voxel in each optical section of the entire image stack was measured as integrated density for the terminals of each LC4 or LPLC2 cluster (max. two per animal, one on each side of the brain).

Imaging Drep2 puncta in GF dendrites was conducted with a Zeiss LSM 980 confocal laser scanning microscope with Airyscan 2 with a 40x water lens, N/A 1.2. Alexa 488 was excited at 488 nm and detected between 510 and 544 nm, Alexa 594 was excited at 594 nm and detected between 597 and 695 nm. For high resolution images, Airyscan 2 was used with a pixel size of 50 × 50 × 190 nm. Images were stored in Zeiss image format. Drep2 puncta were counted using a Python script in Jupyter Notebook (v7.2.2). The script is based on a Blob LoG (Laplacian of Gaussian) algorithm that recognizes round structures whose intensity differs from background. Background was defined by setting a threshold using the Otsu algorithm [89]. For each image stack, a threshold was defined so that no false positives were counted. Local maxima were then counted that coincide with the Drep2 label. Manual inspection of parts of the automated analysis was conducted to ensure proper counts. Co-occurrence with GF dendrites was ensured by using the GFP membrane label of the GF (cd4-td-GFP). Statistical analysis was conducted in GraphPad Prism 10.3.1.

### Visualization of the effectiveness of neuron ablation by expression of UAS-hid under the control of Split-GAL4 drivers

To test how effectively UAS-hid expression ablates neurons, we expressed UAS-hid UAS-stinger and UAS-tdTomato under the control of different Split-GAL4 drivers. UAS-hid and UAS-stinger were on the same chromosome (Bloomington stock #65,408, P{w[+mC]=UAS-Stinger}2, P{w[+mC]=UAS-hid.Z}2/CyO). We tested GF Split GAL4 (+;P{R17A04-p65.AD}attP40 P{w[+mC]=UAS-CD4-tdTom}7M1/CyO;P{R68A06-GAL4.DBD}attP2), PSI-Split GAL4 (w[1118]; P{y[+t7.7] w[+mC]=R10B11-p65.AD}attP40; PBac{y[+mDint2] w[+mC]=UAS-CD4-tdTom}VK00033 P{y[+t7.7] w[+mC]=R13C08-GAL4.DBD}attP2) – kindly provided by Erica Ehrhardt, University of Cologne, Germany; [90]), DLMn-Split GAL4 (Bloomington stock #602,185, w[*]; P{y[+t7.7] w[+mC]=R23H06-p65.AD}attP40, P{w[+mC]=UAS-CD4-tdTom}7M1/CyO; P{y[+t7.7] w[+mC]=R30A07-GAL4.DBD}attP2, [91]), and LC4-Split GAL4 (3R47H03_p65ADZp (attP40); JRC_SS00315 R72E01_ZpGdbd (attP2) – [33]).

Successful ablation was visualized by expression of UAS-tdTomato in the respective neurons.

#### DLMn and PSI.

After dissection of the VNC (see above), specimens were fixed with 4% paraformaldehyde for 50 min, washed 3 × 20 min with PBS, mounted in vectashield (Biozol, Germany, Cat# H-1000) and viewed on a Leica TCS SP8 confocal microscope with a 20x glycerol lens N/A 0.75. TdTomato was visualized with a 561 nm DPSS laser and detected between 565 and 630 nm, GFP was visualized with a 488 nm argon laser and detected between 495 and 540 nm, z-size was 1 µm. Voxel size was 0.568 × 0.568 × 1 µm.

#### GF and LC4.

After dissection of the brain (see above), specimens were treated as for GF, LC4, and LPLCs immunohistochemistry (see above). Primary antibodies were polyclonal chicken IgY α-GFP, 1:1000, abcam ab13970, monoclonal rat α-mCherry, 1:2500, clone 16D7, Invitrogen M11217 and secondary antibodies were goat α-chicken IgY Abberior Star RED, Abberior, STRED-1005, and goat α-rat Alexa 555 (Life Technologies A21434) both 1:500 in 0.1% PBS-Tx. After antibody treatment, specimens were dehydrated with an ascending ethanol series 50, 70, 90, and 100% EtOH for 10 min each, and then mounted in methylsalicylate. Specimen were viewed on a Leica TCS SP8 confocal microscope with a 40x lens, N/A 1.3. Alexa Fluor 555 was visualized with a 561 nm DPSS laser and detected between 565 and 605 nm, Abberior Star RED was visualized with a 633 nm helium neon laser and detected between 645 and 700 nm. Voxel size was 0.284 × 0.284 × 1 µm.

### Electrophysiology

#### GF circuit analysis.

To prepare flies for in vivo extracellular GF stimulation with simultaneous EMG recordings from fiber 6 of the dorsal longitudinal flight muscle (DLM) or the trochanteral jump muscle (TTM), animals were cold anaesthetized (~20 s in an empty pre-chilled 68 ml plastic food vial on ice), transferred to a cold plate (−2 to −3 °C), and glued between head and dorsal thorax to a triangle-bent metal wire (0.1 mm diameter). After that, animals were allowed to recover for 60 min before recording.

Electrophysiological recordings and stimulation in the GF escape circuit were performed with sharpened tungsten wires as previously described [59,92]. Briefly, for GF stimulation, noninsulated, electrolytically sharpened tungsten wires (diameter 100 µm) were placed into the eyes and used as stimulation electrodes. Electrolytical sharpening is done by repeated dipping of the tungsten tip into a NaNO_2_ KOH solution (10.3 M NaNO_2_ and 6.05 M KOH in distilled H_2_O). A similar tungsten wire was placed as a reference electrode into the abdomen. Square pulse stimuli of 0.15 ms duration were delivered with a Grass S88 stimulator (Grass Technologies). Depending on the stimulation amplitude, the GF circuit can be stimulated at two different sites as indicated in the circuit diagram in Fig 2A. High amplitude stimuli (here 15–20 V) cause the initiation of an action potential (AP) in the GF descending neuron. This AP then propagates along the GF axon into the VNC where it is transmitted via 1 electrochemical and 2 chemical synapses onto the fibers of the DLM muscles or via 1 electrochemical synapse onto the TTM, where it causes a SLR that can be read-out by a tungsten recording from a DLM fiber or the TTM (Fig 2A). Alternatively, lower amplitude stimulation (here 5–9 V) does not directly affect the GF descending neuron, but instead, excites upstream photoreceptors and/or first order visual interneurons. The signal is then transmitted through multiple synapses in visual neuropils onto the visual projection neurons LC4 and LPLC2, which in turn synapse onto the GF dendrites (Fig 2A) and cause AP initiation in the GF descending neuron. GF action potentials can then be read-out by a tungsten recording from a DLM/TTM fiber as described for SLR above. However, upon lower amplitude stimulation, synaptic transmission across multiple visual neuropils requires additional time, and thus, can be read-out as LLR from the DLM/TTM. Consequently, changes in SLR are indicative of circuit changes downstream of AP initiation in the GF, whereas changes in LLR without altered SLR are indicative for circuit changes upstream of AP initiation in the GF.

For DLM Mn stimulation, the ventral thorax was penetrated to place the tips of electrolytically sharpened tungsten wires into the ventral nerve cord (VNC) close to the DLMms. Direct electrical stimulation of the DLM Mn can also be read-out DLM target muscle fiber recordings, but it bypasses all GF circuit elements upstream of the motoneurons.

Output of the GF circuit (no matter whether it is stimulated at the level of the visual system, the GF descending neuron, or the motoneurons) can be read-out by an extracellular tungsten recording of any of the DLM fibers [59,92]. Each DLM Mn receives the same input from the GF circuit. Each DLM fiber is innervated by one DLM Mn and each spike recorded from a DLM fiber is a 1:1 reflection of an AP in the DLM Mn innervating that fiber [91]. To test the GF circuit in animals of different ages, we measured SLR, LLR, and DLM responses to direct DLM Mn stimulation. In addition, for SLR we measured refractory period (RP) as the minimal time at which two consecutive stimuli elicit a DLM response as well as following frequency 50% (FF50) as the highest stimulation frequency at which 50% of the stimuli get transmitted through the GF circuit to the DLM fiber. For LLR we also measured habituation [57,58] as the response probability over 1,000 consecutive stimuli given at either 5 Hz or 10 Hz. Alternatively, habituation was quantified by testing how many consecutive stimuli (presented either at 5 Hz or at 10 Hz) were required to induce either 1, 2, 3, 4, or 5 consecutive response failures.

All DLM recordings were acquired in the frequency range of 300 Hz to 10 kHz and amplified 100 times with a differential AC amplifier (A-M Systems model 1,700). Analog output from the amplifier was digitized without further filtering with a Digidata 1,200 AD converter (Molecular Devices) and analyzed with pClamp (version 8.1) software (Molecular Devices).

#### Electroretinogram (ERG) recordings.

The ERG is a compound field potential comprising the light induced sum depolarization of photoreceptors (PRs) as well as electrical responses of first order visual interneurons, lamina monopolar cells (LMCs). ERGs allow a gross assessment of phototransduction as well as synaptic transmission between PRs and LMCs [37,38]. ERG responses are induced by white light stimuli presented in an otherwise dim light background. ERGs are recorded extracellularly with glass microelectrodes filled with 2M KCl inserted just below the cornea of the complex eye and a reference electrode into the abdomen. Extracellular recording, digitization, and data acquisition configuration were as for DLM fiber recordings (see above). For ERG recordings animals were briefly cold anesthetized (~20–30 s in an empty 68 ml plastic food vial on ice) and transferred under a dissection scope. Wings and legs were removed, and the animals were fixated under a fixed stage microscope (Zeiss Axioscope 2 FS plus) in front of a small white light bulb that is controlled by a Grass S88 stimulator (Grass Technologies).

### Behavioral analyses

#### Light-induced escape response (LIER).

LIER was tested by placing single flies in a 9 x 2 cm Petri dish illuminated by LEDs (with peak wavelength at 572 nm). After 15 min accommodation time, 10 lights-off stimuli (1 per minute) of 50 ms duration were provided. A fly was scored a LIER responder upon one successful escape.

#### Optogenetically-induced escape response.

For optogenetic induction of escape UAS-CsChrimson (BDSC#55,136) was expressed at either of three different levels of the GF circuit by using the following neuron-type specific split-GAL4 fly strains. First, [17A04_p65ADZp (attp40); 68A06_ZpGdbd (attP2)] [36] was used for selective expression of CsChrimson in the GF descending neuron. Second, R47H03_p65ADZp (attP40); JRC_SS00315 R72E01_ZpGdbd (attP2) was used for selective expression of CsChrimson in LC4 visual projection neurons, and third, R19G02_p65ADZp (attP40); R75G12_ZpGdbd (attP2) for expression in LPLC2 visual projection neurons [87]. As control UAS-GFP was expressed instead of UAS-CsChrimson under the control of the same split-GAL4 drivers. For optogenetic stimulation, individual flies were placed in a 9 × 2 cm Petri dish, viewed through an infrared camera under dim infrared light illumination, and then stimulated three times (every 10 s) with red-light pulses (594 nm) of 2 s duration. An animal was scored as responder upon responding with escape to at least one of the three consecutive red-light pulses.

### Starvation assay

Five days old flies of both sexes were singled out in vials with 0.5% agar to undergo starvation. Flies were tested every three hours until death for escape performance to mechanical stimulation in the startle assay as previously described [25]. Flies that did not respond to 5 consecutive startle stimuli were defined as NRs, whereas escape in response to at least one of three startle stimuli was defined as responder. Hyperactivity periods (intense walking and/or climbing activity as indicative in searching for food) intermingled with standing pauses were common in starved individuals. However, as starved flies approach the time of death, they often exhibit signs of functional collapse as previously described [25]. Re-feeding successfully rescued starvation induced nonresponsiveness as well as other pre-death motor symptoms. A longitudinal assay with a temporal resolution of 3 hours was conducted with 38 male flies from starvation start at 5 days until death. In addition, starvation and re-feeding were used to select animals for electrophysiological testing of the GF circuit in young responders and young NRs.

### Oxidative stress assay

Five days old flies were starved for 7 hours and then transferred to vials with filter paper soaked in 1% Sucrose and 1% H_2_O_2_ [93]. Lethality was observed within 2–4 days with males dying first. Flies were tested every three hours until death for escape performance to mechanical stimulation as in the starvation assay. Electrophysiological recordings were performed in responders and NRs of the same age.

### Connectomics analysis

Both the Janelia hemibrain connectome v1.2.1 [44] and the Male Adult Ventral Nerve Cord (MANC) connectome [53] were accessed via the Python neuPrint API [94] and analyzed using the Neuron Analysis and Visualisation (NAVis) library (v1.10.0, [95]). The Janelia hemibrain dataset contains reconstructions, synapses and annotations of neurons of central brain neuropils, optic lobe neuropils as well as the left giant fiber descending neuron (GF). Cell types of all GF presynaptic input partners were analyzed to input area and how many input synapses they contribute to the GF visual dendrites. The MANC dataset, which encompasses a complete reconstruction of a male fly ventral nerve cord, was used to assess the number and localization of input synapses to the DLM Mns as part of the GF escape circuit both by the peripherally synapsing interneuron (PSI) as well as by a candidate alternative route, the giant fiber coupled neurons 2 (GFC2, [54]).

The Full Adult Fly Brain (FAFB) connectome v783 [55] is as of now not publically accessible via Python APIs, but annotated connectivity data [96–98], can be downloaded via codex.flywire.ai/api/download?dataset = fafb [99]. We used the synaptic connectivity table (Version 20.05.2025, updated by Princeton) as well as consolidated cell type annotations (Version 08.06.2025). To analyze shortest paths from all photoreceptors R1-6, R7, and R8 to the GF, data was first sorted to only contain the right brain hemisphere and further restricted to only contain connections of 5 or more synapses. Using the connectivity data table, a directed graph with edge weights between neurons representing the number of synapses was created using the NetworkX Python package v3.5 [100]. For each R1-6, R7, and R8 photoreceptor the shortest paths to the GF were calculated and in case of multiple shortest paths only those with the highest total synapse count were kept. With these strongest of the shortest pathways, Sankey diagrams were plotted using the Plotly Open-Source Graphing Libraries, with nodes representing cell types along pathways and edge thickness representing the total count of individual connections between cell types.

### Statistical analysis

Statistical analysis was performed using GraphPad Prism version 6.00 for Windows (GraphPad Software, La Jolla California USA, www.graphpad.com) and SPSS statistics. Normally distributed data (assessed with normality D’Agostino &Pearson omnibus normality test) are graphed as means and SEM. Student’s *t* test was used to test for statistically significant differences between two groups and ANOVA with Tukey and/or LSD post hoc comparison for planned statistical comparison of more than two test groups. Fisher’s exact test was used to test counts in categories in contingency tables. Significance levels were defined as *p < 0.05; ** *p* < 0.01, ***p < 0.001).

## Supporting information

S1 Data Contains all values used for data presentation and statistical analysis.
(XLSX)

S1 Fig Abolishment of neurons by UAS-hid expression is differentially effective. UAS-hid was expressed in different neurons under the control of Split-GAL4 drivers. Co-expression of UAS-tdTomato allowed for visualization of the neurons while co-expression of UAS-stinger on the same chromosome as UAS-hid enabled visualization of expression of UAS-transgenes off that chromosome in the respective animal. (A–H) UAS-hid expression under the control of GF-Split-GAL4 ablated one GF in 50% of the cases, while in the other 50% both GFs remained. The GF was visualized in the brain (A-C, E-G) and in the VNC (D, H). UAS-tdTomato (A, D, E, H, magenta) and UAS-stinger (B, C, F, G, green) are expressed. (I, J) UAS-tdTomato was expressed under the control of DLMn-Split-GAL4 DLMNs in the absence (I) and in the presence of UAS-Hid (J). Expression of UAS-hid reliably ablates DLMns (J). Laser intensity was increased strongly to be able to see the outline of the VNC in the absence of tdTomato label (J). (K–N) UAS-hid expression under the control of PSI-Split-GAL4 ablated both PSI in 75% of the cases, while in the other 25% both PSI remained. PSI neurons are shown in magenta (K, M), UAS-stinger is shown in green (L). (O and P). Expression of UAS-hid in LC4 neurons almost completely ablates these neurons. Of the normally ~75 neurons per hemisphere (O, green), only a few remain after expression of UAS-hid and UAS-tdTomato (P, R) and UAS-stinger (Q, green). (S) Quantification of ablation efficiency by expression of UAS-hid under the control of different Split-GAL4 drivers. GF: left bar, black/light gray. Expression under the control of GF Split-GAL4 never ablated both GF descending neurons. Rather, in 50% of the animals both GF neurons remained (black bar), while in the other 50% one GF was ablated, amounting to an efficiency of 25% ablation if UAS-hid is expressed (light gray bar on the left). DLM Mn: second bar gray, all neurons ablated; PSI: third bar, dark gray, if ablated, then both neurons are gone; LC4 Split-GAL4: right bar, light gray, almost all neurons ablated. See S1 Data for individual data points.
(TIF)

S2 Fig SLR and LLR read-outs from the DLM in responders and NRs after exposure to oxidative stress. Animals were exposed to oxidative stress and then separated by behavioral testing into two groups, responders (black bars) and NRs (red bars). Bars indicate the mean values for the short latency response (SLR, left), the refractory period (RP, middle), and the frequency at which 50% of the stimuli resulted in a read-out (following frequency 50, FF50, right). The SLR was reliably present in both groups and not significantly different between responders and NRs (unpaired Student’s test, *p* = 0.072). By contrast, the LLR was normal in responders but absent in NRs. Both additional measures taken from SLR read-outs, RP and FF50 were not significantly different in responders and NRs (unpaired Student’s *t* tests, *p* = 0.6 for RP and 0.21 for FF50). See S1 Data for individual data points.
(TIF)

S3 Fig GF visual dendrite with postsynaptic marker expression in responders versus NRs. Maximum intensity projection views from confocal image stacks taken from the GF visual dendrites with expression of UAS-GFP (green, Ai to Ji) and with expression of Drep2, a marker for postsynaptic cholinergic sites (magenta, Aii to Jii) for 5 responding (A–E) and for 5 nonresponding animals (F–J). Scale bar is 5 µm.
(TIF)

S4 Fig LPLC2 neurons with expression of the active zone marker BRP.S in responders versus NRs. Maximum intensity projection views from confocal image stacks taken from the LPLC2 visual projection neurons with expression of UAS-GFP (green) and the active zone marker UAS-BRP-S (magenta). Shown are overlay images of both color channels, overlap of green and magenta in the synaptic terminals appears white. In addition, white overlap label is detected in the somata of the LPLC2 neurons where the protein is synthesized. This made somata counts for Fig 7N easier. The top row shows image stacks taken from >60 days old responders, whereas the bottom row shows image stacks taken from >60 days old NRs.
(TIF)

S5 Fig LC4 neurons with expression of the active zone marker BRP.S in responders versus NRs. Maximum intensity projection views from confocal image stacks taken from the LC4 visual projection neurons with expression of UAS-GFP (green) and the active zone marker UAS-BRP-S (magenta). Shown are overlay images of both color channels, overlap of green and magenta in the synaptic terminals appears white. In addition, white overlap label is detected in the somata of the LC4 neurons where the protein is synthesized. This made somata counts for Fig 7K easier. The top row shows image stacks taken from >60 days old responders, whereas the bottom row shows image stacks taken from >60 days old NRs.
(TIF)

## Abbreviations

AD: Alzheimer’s disease
AP: action potential
CLSM: confocal laser scanning microscopy
Cij: one-way mixing effect
DLM: dorsal longitudinal flight muscle
ERGs: electroretinograms
FAFB: Full Adult Fly Brain
GF: giant fiber
GFC2: giant fiber coupled neurons 2
GFS: giant fiber system
IIS: insulin-like signaling
LC4: lobular columnar type 4
LMCs: lamina monopolar cells
LIER: Light-induced escape response
LLR: long latency response
LPLC2: lobular plate/lobular columnar type 2
MANC: Male Adult Ventral Nerve Cord
NAVis: Neuron Analysis and Visualisation
PBS: phosphate buffered saline
PRs: photoreceptors
PSI: peripherally synapsing interneuron
RP: potential/photoreceptor potential
SLR: short latency response
TTM: trochanteral jump muscle
TTM Mn: TTM jumping motoneuron
VNC: ventral nerve cord
