# Supplementary figures and images for: The Drosophila escape motor circuit shows differential vulnerability to aging linked to functional decay
Source: PLoS Biol. 2025 Dec 16;23(12):e3003553. doi: 10.1371/journal.pbio.3003553 (PMC12707685; doi:10.1371/journal.pbio.3003553)

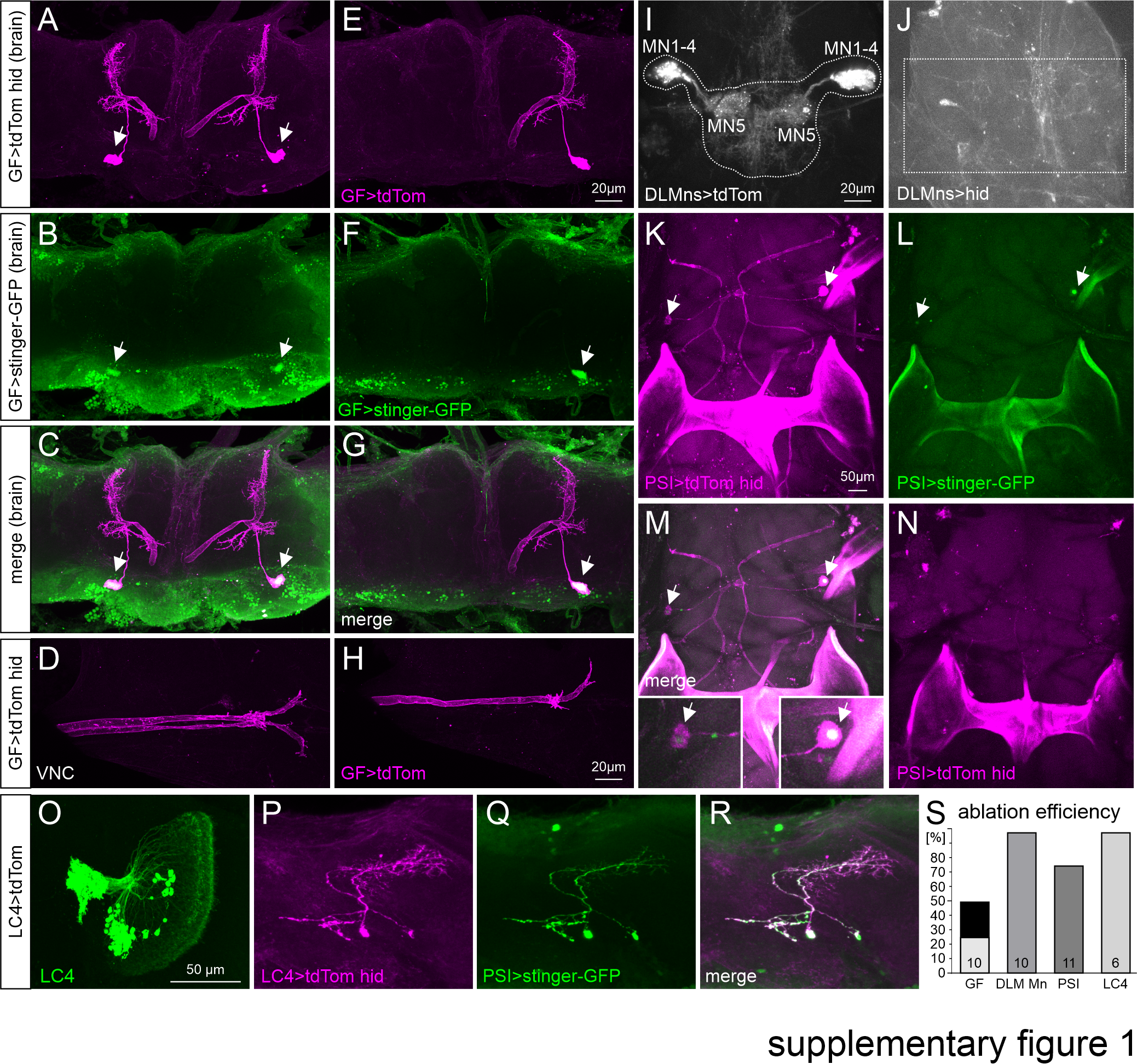

Supplement: S1 Fig — Abolishment of neurons by UAS-hid expression is differentially effective. UAS-hid was expressed in different neurons under the control of Split-GAL4 drivers. Co-expression of UAS-tdTomato allowed for visualization of the neurons while co-expression of UAS-stinger on the same chromosome as UAS-hid enabled visualization of expression of UAS-transgenes off that chromosome in the respective animal. (A–H) UAS-hid expression under the control of GF-Split-GAL4 ablated one GF in 50% of the cases, while in the other 50% both GFs remained. The GF was visualized in the brain (A-C, E-G) and in the VNC (D, H). UAS-tdTomato (A, D, E, H, magenta) and UAS-stinger (B, C, F, G, green) are expressed. (I, J) UAS-tdTomato was expressed under the control of DLMn-Split-GAL4 DLMNs in the absence (I) and in the presence of UAS-Hid (J). Expression of UAS-hid reliably ablates DLMns (J). Laser intensity was increased strongly to be able to see the outline of the VNC in the absence of tdTomato label (J). (K–N) UAS-hid expression under the control of PSI-Split-GAL4 ablated both PSI in 75% of the cases, while in the other 25% both PSI remained. PSI neurons are shown in magenta (K, M), UAS-stinger is shown in green (L). (O and P). Expression of UAS-hid in LC4 neurons almost completely ablates these neurons. Of the normally ~75 neurons per hemisphere (O, green), only a few remain after expression of UAS-hid and UAS-tdTomato (P, R) and UAS-stinger (Q, green). (S) Quantification of ablation efficiency by expression of UAS-hid under the control of different Split-GAL4 drivers. GF: left bar, black/light gray. Expression under the control of GF Split-GAL4 never ablated both GF descending neurons. Rather, in 50% of the animals both GF neurons remained (black bar), while in the other 50% one GF was ablated, amounting to an efficiency of 25% ablation if UAS-hid is expressed (light gray bar on the left). DLM Mn: second bar gray, all neurons ablated; PSI: third bar, dark gray, if ablated, then both neurons a [file pbio.3003553.s002.tif]

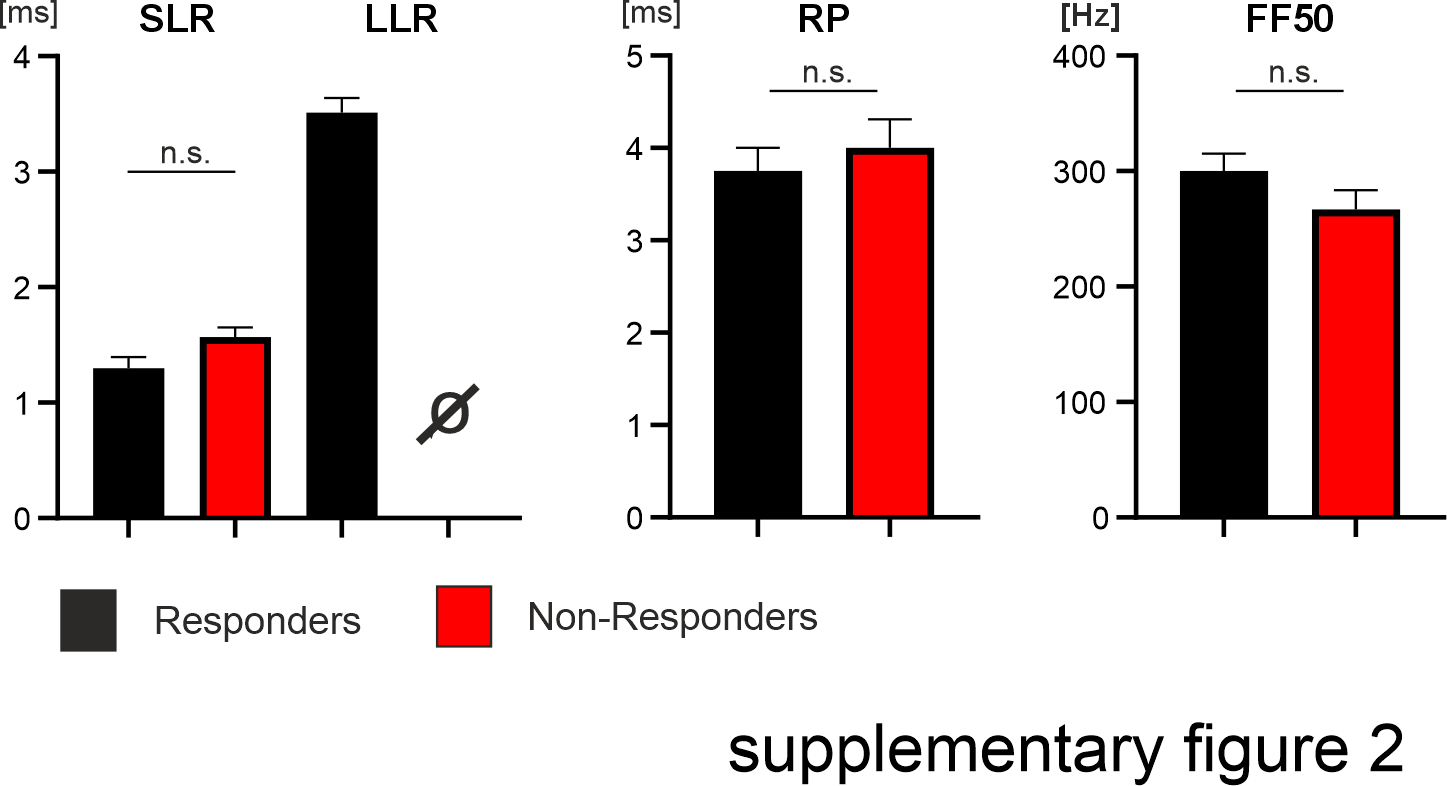

Supplement: S2 Fig — SLR and LLR read-outs from the DLM in responders and NRs after exposure to oxidative stress. Animals were exposed to oxidative stress and then separated by behavioral testing into two groups, responders (black bars) and NRs (red bars). Bars indicate the mean values for the short latency response (SLR, left), the refractory period (RP, middle), and the frequency at which 50% of the stimuli resulted in a read-out (following frequency 50, FF50, right). The SLR was reliably present in both groups and not significantly different between responders and NRs (unpaired Student’s test, p = 0.072). By contrast, the LLR was normal in responders but absent in NRs. Both additional measures taken from SLR read-outs, RP and FF50 were not significantly different in responders and NRs (unpaired Student’s t tests, p = 0.6 for RP and 0.21 for FF50). See S1 Data for individual data points. (TIF) [file pbio.3003553.s003.tif]

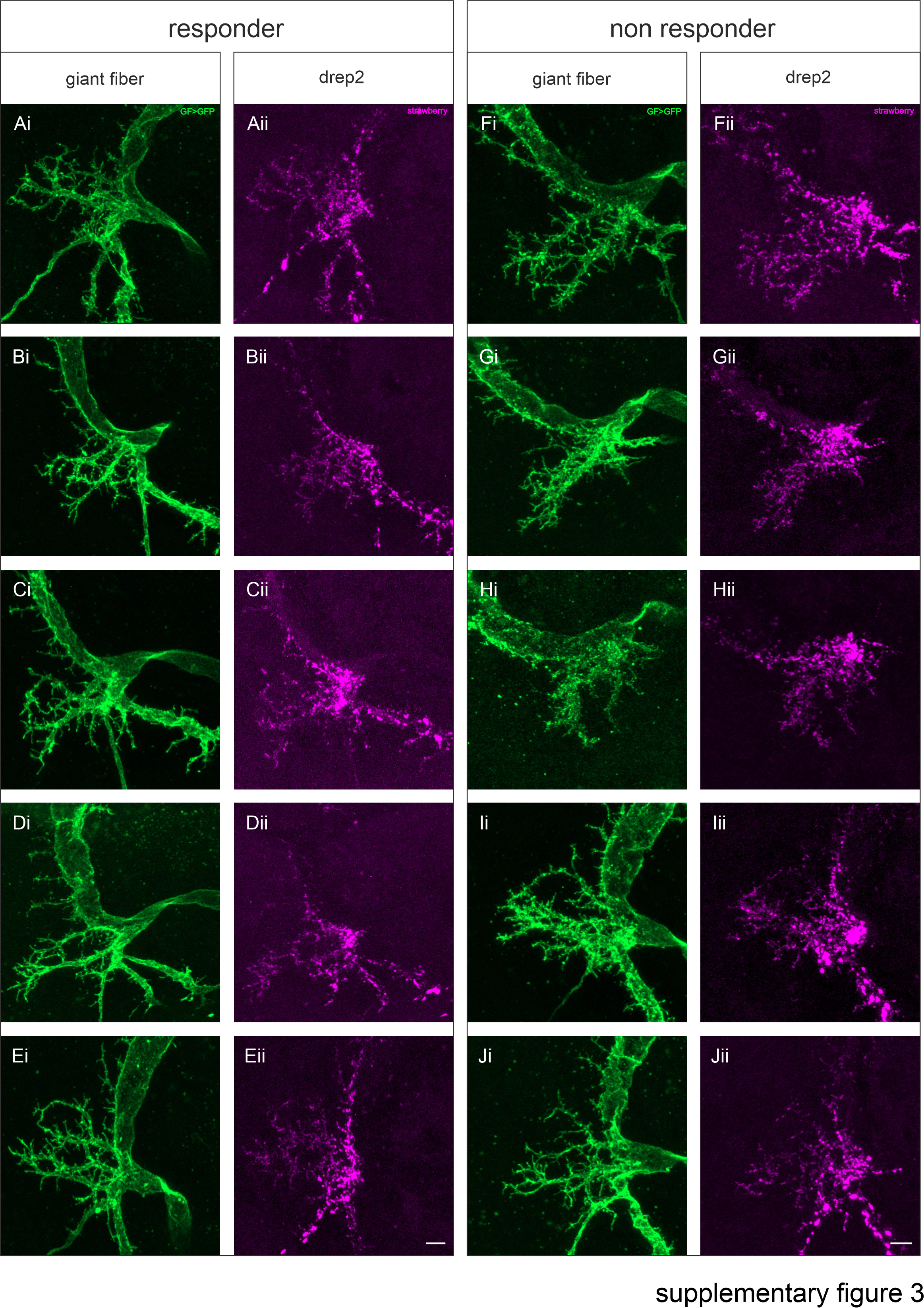

Supplement: S3 Fig — GF visual dendrite with postsynaptic marker expression in responders versus NRs. Maximum intensity projection views from confocal image stacks taken from the GF visual dendrites with expression of UAS-GFP (green, Ai to Ji) and with expression of Drep2, a marker for postsynaptic cholinergic sites (magenta, Aii to Jii) for 5 responding (A–E) and for 5 nonresponding animals (F–J). Scale bar is 5 µm. (TIF) [file pbio.3003553.s004.tif]

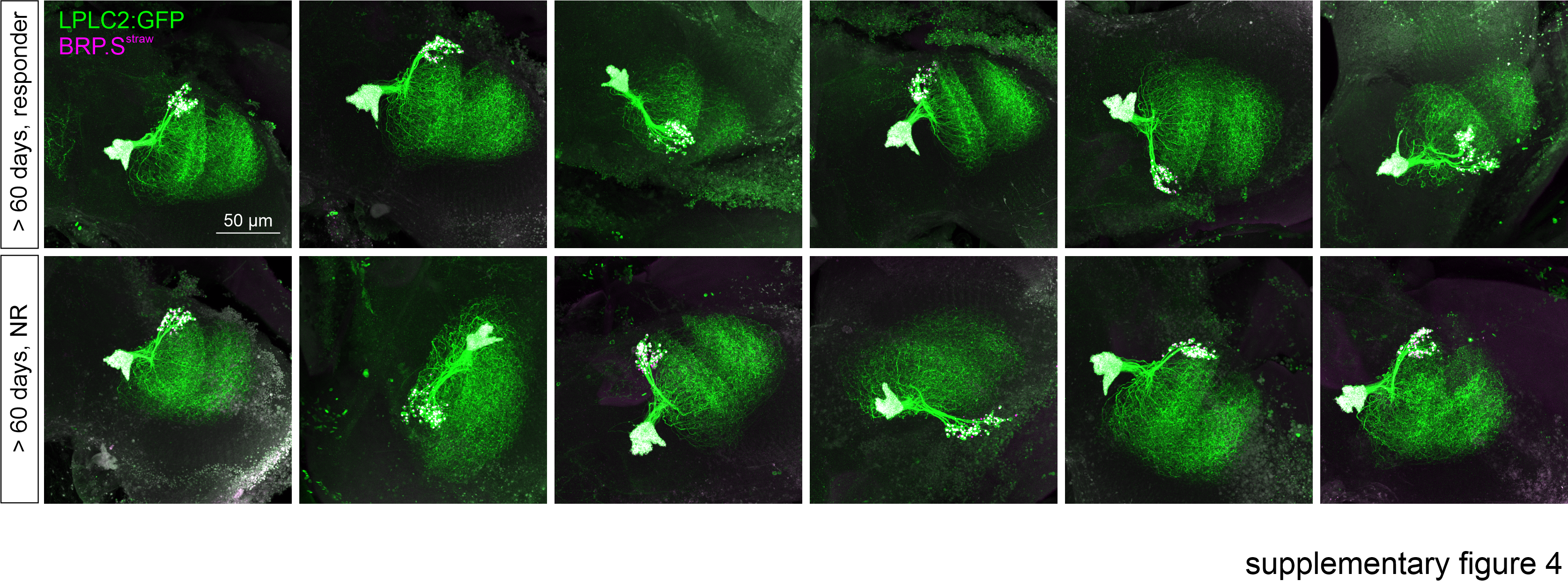

Supplement: S4 Fig — LPLC2 neurons with expression of the active zone marker BRP.S in responders versus NRs. Maximum intensity projection views from confocal image stacks taken from the LPLC2 visual projection neurons with expression of UAS-GFP (green) and the active zone marker UAS-BRP-S (magenta). Shown are overlay images of both color channels, overlap of green and magenta in the synaptic terminals appears white. In addition, white overlap label is detected in the somata of the LPLC2 neurons where the protein is synthesized. This made somata counts for Fig 7N easier. The top row shows image stacks taken from >60 days old responders, whereas the bottom row shows image stacks taken from >60 days old NRs. (TIF) [file pbio.3003553.s005.tif]

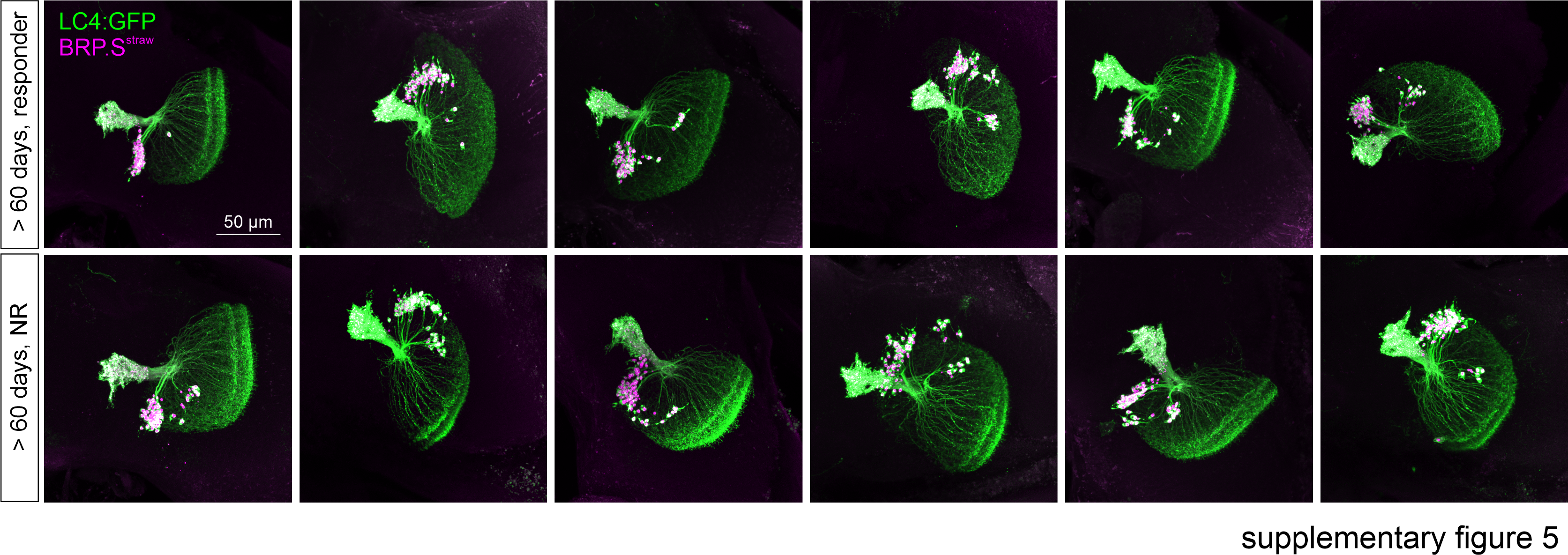

Supplement: S5 Fig — LC4 neurons with expression of the active zone marker BRP.S in responders versus NRs. Maximum intensity projection views from confocal image stacks taken from the LC4 visual projection neurons with expression of UAS-GFP (green) and the active zone marker UAS-BRP-S (magenta). Shown are overlay images of both color channels, overlap of green and magenta in the synaptic terminals appears white. In addition, white overlap label is detected in the somata of the LC4 neurons where the protein is synthesized. This made somata counts for Fig 7K easier. The top row shows image stacks taken from >60 days old responders, whereas the bottom row shows image stacks taken from >60 days old NRs. (TIF) [file pbio.3003553.s006.tif]
